# Clinical significance of *FAT1* gene mutation and mRNA expression in patients with head and neck squamous cell carcinoma

**DOI:** 10.1002/1878-0261.13171

**Published:** 2022-01-13

**Authors:** Su Il Kim, Seon Rang Woo, Joo Kyung Noh, Min Kyeong Lee, Young Chan Lee, Jung Woo Lee, Seong‐Gyu Ko, Young‐Gyu Eun

**Affiliations:** ^1^ Department of Biomedical Science and Technology Graduate School Kyung Hee University Seoul Korea; ^2^ Department of Otolaryngology‐Head and Neck Surgery Kyung Hee University Medical Center Seoul Korea; ^3^ Department of Oral and Maxillofacial Surgery School of Dentistry Kyung Hee University Seoul Korea; ^4^ Department of Preventive Medicine College of Korean Medicine Kyung Hee University Seoul Korea

**Keywords:** FAT1, gene signature, head and neck squamous cell carcinoma, overall survival, recurrence‐free survival

## Abstract

The *FAT1* gene functions as a tumor suppressor or promoter and remains incompletely understood. We examined the clinical significance of *FAT1* in head and neck squamous cell carcinoma (HNSCC) using four publicly available HNSCC cohorts and one HNSCC cohort enrolled at a tertiary medical center. We developed *FAT1* signatures reflecting *FAT1* mutations and mRNA expression using one cohort. Patients with HNSCC were classified into *FAT1*‐associated low risk (FAT1‐LR; *n* = 195) and *FAT1*‐associated high risk (FAT1‐HR; *n* = 371) subgroups. The five‐year overall survival and recurrence‐free survival rates were significantly lower in the FAT1‐HR subgroup than in the FAT1‐LR subgroup (*P* = 0.01 and 0.003, respectively). The clinical significance of *FAT1* was validated using four independent cohorts. Cox proportional hazards models showed that the *FAT1* signature was an independent prognostic factor for HNSCC patients. In addition, *FAT1* signature was associated with the response to radiotherapy, advanced stage, and human papilloma virus (HPV) status in HNSCC patients. In conclusion, the *FAT1* gene signature was associated with prognosis of HNSCC and may help to provide personalized treatments for HNSCC patients.

## Graphical abstract

In our study, we classified patients with head and neck squamous cell carcinoma (HNSCC) as FAT1‐associated low risk and FAT1‐associated high risk, based on our predefined FAT1 gene‐related molecular signature. The prognosis of patients with HNSCC was significantly different between both subgroups. In conclusion, the FAT1 signature served as an independent prognostic factor for HNSCC and was associated with response to radiation therapy.

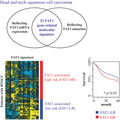

**Abbreviations**
ATCC: American Type Culture Collection
AUC: area under the curve
BCCP: Bayesian compound covariate predictor
CFA: colony‐forming assay
ddPCR: droplet digital PCR
DMEM: Dulbecco’s modified Eagle’s medium
FAT1‐HR: FAT1‐associated high risk
FAT1‐LR: FAT1‐associated low risk
FC: fold change
GFP: green fluorescent protein
HNSCC: head and neck squamous cell carcinoma
HPV: human papilloma virus
HR: hazard ratio
ICGC: International Cancer Genome Consortium
JCRB: Japanese Cancer Resources Bank
KCLB: Korean Cell Line Bank
KHUMC: Kyung Hee University Medical Center
OS: overall survival
PI: propidium iodide
PS: penicillin–streptomycin
RFS: recurrence‐free survival
STR: short tandem repeat
TCGA: The Cancer Genome Atlas

## 1 Introduction

Head and neck squamous cell carcinoma (HNSCC) is the sixth most common cancer worldwide and includes all cancers that occur in the mucosa of the oral cavity, oropharynx, larynx, and hypopharynx [1]. It is known that 650 000 new HNSCC patients occur and 350 000 die worldwide every year [2]. Despite the improved quality of life in patients with HNSCC, due to advancements in treatment modalities such as advanced surgical technique and radiotherapy, the survival rates of this condition have not markedly improved in recent decades [3]. The HPV status is well‐known factor influencing prognosis of patients with HNSCC [4, 5]. Recently, various molecular markers that influence prognosis of patients with HNSCC are being actively identified for precision medicine and personalized treatment [6, 7]. However, there are still many other molecular markers that need to be researched.

The human FAT1 gene encodes a large transmembrane protein with extracellular Cadherin repeats, EGF‐like domains, and Laminin G‐like domains that is commonly expressed in epithelial tissues [8, 9]. Binding of Ena/VAPS to FAT1 promotes cell migration [10]. On the other hand, binding of Scribble to FAT1 inhibits YAP1‐associated cell proliferation [11]. FAT1 also inhibits Wnt/β‐catenin signaling, which is important for cell proliferation and tumor growth [12]. In other words, the role of FAT1 varies, from promoting cell migration to inhibiting cell proliferation and growth.

The function of FAT1 remains unclear, because of its complexity in modulating tumorigenesis. FAT1 functions as a tumor suppressor or promoter, depending on the variety of cancer. It has been reported that mutation and low expression of FAT1 are important factors in the development of oral cancer [13]. In addition, mutation and low expression of FAT1 are independent predictors of poor prognosis in patients with HNSCC [14]. Inactivation of FAT1 via mutation promotes Wnt signaling and tumorigenesis at various sites [15]. However, in a recent study, FAT1 mutation was found to be significantly associated with better prognosis in HPV‐negative [HPV (−)] HNSCC patients [16]. In addition, patients with high expression of FAT1 displayed worse prognosis in HNSCC [15, 17]. Therefore, additional studies are required to validate the role of FAT1 in HNSCC and to determine personalized treatment for patients with HNSCC.

We aimed to systemically analyze the genomic data of patients with HNSCC, to determine the molecular subtypes associated with FAT1 and prognosis of patients with HNSCC. We hypothesized that investigation of FAT1 gene expression and mutation data in The Cancer Genome Atlas (TCGA) HNSCC data would help generate FAT1‐related molecular signatures, which could be validated in various independent HNSCC cohorts. We also investigated the prognostic importance of FAT1 signatures in various subgroups of patients with HNSCC.

## 2 Materials and methods

### 2.1 Patient cohorts

Gene expression levels and clinical data from four independent cohorts were downloaded from public databases. Gene expression and clinical data from the TCGA cohort (*n* = 566) were downloaded from the UCSC Cancer Genomics Browser (https://xena.ucsc.edu/public). Data from the Institute for Medical Informatics, Statistics and Epidemiology (Leipzig cohort, GSE65858, *n* = 270), Fred Hutchinson Cancer Research Center (FHCRC cohort, GSE41613, *n* = 97), and MD Anderson Cancer Center (MDACC cohort, GSE42743, *n* = 74) were downloaded from the National Center for Biotechnology Information Gene Expression Omnibus database (http://www.ncbi.nlm.nih.gov/geo). The gene expression profile of the TCGA cohort was measured using Illumina HiSeq 2000 (Illumina Inc., San Diego, CA, USA), that of the Leipzig cohort using Illumina HumanHT‐12 v4.0 Expression Beadchip, and those of the FHCRC and MDACC cohorts using Affymetrix Human Genome U133 Plus 2.0 Array (Affymetrix Inc., Santa Clara, CA, USA).

### 2.2 KHUMC patient dataset

Patients diagnosed with HNSCC at the Kyung Hee University Medical Center (KHUMC) were enrolled in this study between Jan 2011 and Jan 2019. A total of seventy‐two patients who received curative management and underwent genetic analysis were prospectively followed up upon (KHUMC cohort, *n* = 72). Patients with HNSCC as a second primary, a history of prior treatment for a malignant tumor of any head or neck site, or tumors with nonsquamous cell carcinoma histological characteristics were excluded from this study. Clinical data of patients, including age, sex, smoking history, and therapeutic type, were reviewed. In addition, survival and recurrence of patients were retrospectively checked for 5 years after treatment. Age was categorized as < 60 years or ≥ 60 years. Smoking history was categorized as never or ever. T‐ and N‐staging was based on the American Joint Committee on Cancer Staging Manual, Seventh Edition (AJCC) [18], and categorized as T1–T2 or T3–T4 and N0 or N1‐3, respectively, for statistical analysis (Table 1).

**Table 1:** Clinical and pathological characteristics of the five independent HNSCC cohorts. FAT1‐HR, FAT1‐associated high risk; FAT1‐LR, FAT1‐associated low risk; FHCRC, Fred Hutchinson Cancer Research Center; HNSCC, head and neck squamous cell carcinoma; KHUMC, Kyung Hee University Medical Center; MDACC, MD Anderson Cancer Center; NA, not available; TCGA, The Cancer Genome Atlas.

Characteristics	TCGA cohort (*n* = 566)	Leipzig cohort (*n* = 270)	FHCRC cohort (*n* = 97)	MDACC cohort (*n* = 74)	KHUMC cohort (*n* = 72)
Age					
≥ 60	316 (55.83%)	117 (43.33%)	47 (48.45%)	37 (50.00%)	44 (61.11%)
< 60	249 (43.99%)	153 (56.67%)	50 (51.55%)	37 (50.00%)	27 (37.50%)
Unknown	1 (0.18%)	0	0	0	1 (1.39%)
Sex					
Male	415 (73.32%)	223 (82.59%)	66 (68.04%)	58 (78.38%)	54 (75.00%)
Female	151 (26.68%)	47 (17.41%)	31 (31.96%)	16 (21.62%)	18 (25.00%)
Smoking					
Yes	425 (75.09%)	222 (82.22%)	NA	59 (79.73%)	45 (62.50%)
No	128 (22.61%)	48 (17.78%)	NA	15 (20.27%)	25 (34.72%)
Unknown	13 (2.3%)	0	NA	0	2 (2.78%)
Alcohol					
Yes	371 (65.55%)	239 (88.52%)	NA	NA	NA
No	182 (32.16%)	31 (11.48%)	NA	NA	NA
Unknown	13 (2.3%)	0	NA	NA	NA
Tumor site					
Oral cavity	346 (61.13%)	83 (30.74%)	97 (100%)	71 (95.95%)	43 (59.72%)
Oropharynx	82 (14.49%)	102 (37.78%)	0	3 (4.05%)	11 (15.28%)
Larynx	128 (22.61%)	48 (17.78%)	0	0	18 (25.00%)
Hypopharynx	10 (1.77%)	33 (12.22%)	0	0	0 (%)
Unknown	0	4 (1.48%)	0	0	0 (%)
T classification					
T1–T2	218 (38.52%)	115 (42.59%)	NA	30 (40.54%)	33 (45.83%)
T3–T4	344 (60.78%)	155 (57.41%)	NA	44 (59.46%)	30 (41.67%)
Unknown	4 (0.71%)	0	NA	0	9 (12.50%)
N classification					
Negative	295 (52.12%)	94 (34.81%)	NA	42 (56.76%)	35 (48.61%)
Positive	267 (47.17%)	176 (65.19%)	NA	32 (43.24%)	29 (40.28%)
Unknown	4 (0.71%)	0	NA	0	8 (11.11%)
Stage					
I–II	135 (23.85%)	55 (20.37%)	41 (42.27%)	19 (25.68%)	24 (33.33%)
III–IV	417 (73.67%)	215 (79.63%)	56 (57.73%)	55 (74.32%)	30 (41.67%)
Unknown	14 (2.47%)	0	0	0	18 (25.00%)
HPV status					
Positive	21 (3.71%)	60 (22.22%)	0 (%)	NA	3 (4.17%)
Negative	65 (11.48%)	209 (77.41%)	97 (100%)	NA	15 (20.83%)
Unknown	480 (84.81%)	1 (0.37%)	0	NA	54 (75.00%)
Radiotherapy					
Yes	304 (53.71%)	NA	NA	47 (63.51%)	43 (59.72%)
No	171 (30.21%)	NA	NA	26 (35.14%)	27 (37.50%)
Unknown	91 (16.08%)	NA	NA	1 (1.35%)	2 (2.78%)
Treatment					
Unimodal		78 (28.89%)	43 (44.33%)	25 (33.78%)	31 (43.06%)
Multimodal		189 (70.00%)	53 (54.64%)	48 (64.87%)	41 (56.94%)
Palliative		3 (1.11%)	0	0	0
Unknown		0	1 (1.03%)	1 (1.35%)	0
FAT1 signature					
FAT1‐LR	195 (34.45%)	101 (37.41%)	31 (31.96%)	19 (25.68%)	27 (37.5%)
FAT1‐HR	371 (65.55%)	169 (62.59%)	66 (68.04%)	55 (74.32%)	45 (62.5%)

### 2.3 Collection and storage of tissues

Immediately after surgery, tissue samples (surgical specimens or biopsies) of HNSCC patients were collected by healthcare professionals, in tubes containing the stabilizing reagent TRIzol^®^ reagent (Invitrogen, Carlsbad, CA, USA), following which the tubes were quickly placed in a cage filled with liquid nitrogen. The samples were then transported to the patient specimen storage facility; the transport time was < 1 h. Upon arrival, the tissue samples were recorded and promptly stored at −80 °C.

### 2.4 Isolation of nucleic acids, real‐time quantitative reverse transcriptase PCR analysis, quality control check, and transcriptome sequencing

Total RNA was extracted using the TRIzol^®^ Reagent method as previously described [19]. In more detail, frozen tissues were homogenized in 1 mL TRIzol^®^ Reagent and briefly vortexed, following which 200 μL chloroform was added to the samples. The mixtures were centrifuged for 15 min at 12 000 ***g*** at 4 °C, which resulted in them getting separated into a lower red phenol–chloroform interphase and a colorless upper aqueous phase. The supernatant containing the RNA was then transferred to a new tube. RNA precipitation was performed with 500 μL isopropanol, and the RNA pellets were washed with 1 mL of 70% ice‐cold ethanol.

Total RNA was reverse transcribed to cDNA using a Tetro cDNA Synthesis Kit (Bioline, London, UK), according to the manufacturer's recommended protocol. Real‐time quantitative PCR was performed using *the SensiFAST™ SYBR Hi‐ROX Kit* (Bioline) with specific primers. All real‐time quantitative PCR experiments were performed in triplicate, and quantification cycle (Cq) values were determined using stepone software v2.3 (Applied Biosystems, Foster City, CA, USA). Relative quantification of mRNA levels was performed using the comparative Ct method, with β‐actin as the reference gene. The following primer sets were used for real‐time PCR experiments: β‐actin, 5′‐GGACTTCGAGCAAGAGATGG‐3′ (forward) and 5′‐AGCACTGTGTTGGCGTACAG‐3′ (reverse); FAT1, 5′‐ CCTTCCAACAGCCACATCCACTAC‐3′ (forward) and 5′‐TTGAACCGTGAGCGTGTAACCTG‐3′ (reverse). All experiments were performed in triplicate, and the values were averaged.

Isolated total RNA samples were sent to Applied Biosystems Macrogen Korea for sequencing, pre‐processing, and transcriptome analysis. Total RNA concentration was calculated using Quant‐IT RiboGreen^®^ (Invitrogen, #R11490). Only high‐quality RNA preparations, with RIN > 7.0, were used for RNA library construction. A library was independently prepared with 1 μg of total RNA for each sample, using the Illumina TruSeq^®^ Stranded mRNA Sample Prep Kit (Illumina Inc., #RS‐122‐2101). The libraries were quantified using KAPA Library Quantification Kits for Illumina Sequencing platforms, according to the qPCR Quantification Protocol Guide (Kapa Biosystems, Wilmington, MA, USA, #KK4854) and qualified using the TapeStation D1000 ScreenTape (Agilent Technologies, Palo Alto, CA, USA, #5067‐5582). Indexed libraries were then submitted to an Illumina NovaSeq (Illumina Inc.), and paired‐end (2 × 100 bp) sequencing was performed by Macrogen Inc. (Seoul, Korea).

### 2.5 Identification of FAT1 signature

To identify FAT1‐related genes in HNSCC, gene expression and mutation data were analyzed from the TCGA cohort. Fold change (FC) was calculated between the FAT1 mutation and wild‐type groups for every gene, to identify differentially expressed genes. Student's *t*‐test was also used to measure the statistical significance of each gene under the null hypothesis that the gene is not differentially expressed in different samples [20]. Pearson's correlation was used to determine the correlation between the RNA expression levels of FAT1 and other genes. FAT1‐related genes were selected if they satisfied all four inclusion criteria: (1) FC ≥ 1.5 [20], (2) Student *t*‐test *P*‐value <0.05, (3) |Pearson's correlation coefficients R| > 0.2, and (4) Pearson's correlation *P*‐value <0.05. We then performed a hierarchical clustering analysis with the centered correlation coefficient as a measure of similarity and a complete linkage clustering method, using cluster 3.0 (Stanford University, Stanford, CA, USA). Patients were divided into two subgroups, FAT1‐associated low risk (FAT1‐LR) and FAT1‐associated high risk (FAT1‐HR), based on the results of patient‐clustering analysis.

### 2.6 Construction of prediction models and validation in the four independent cohorts

Before constructing the prediction models, all gene expression data for each cohort were standardized, because these were generated using different platforms. The Bayesian compound covariate predictor (BCCP) class prediction engine was adopted to test the ability of gene signatures to predict the class of patients in another four independent cohorts [21]. Gene expression data in the training set (TCGA cohort) were combined to form a series of classifiers according to the BCCP algorithm, and the robustness of the classifier was estimated according to the misclassification rate determined during leave‐one‐out cross‐validation of the training set using BRB–ArrayTools [22]. Validation was conducted in the four independent cohorts (Leipzig, FHCRC, MDACC, and KHUMC).

### 2.7 Statistical analysis

To test prognostic significance, gene expression data with available survival data were used. Overall survival (OS) was defined as the number of months from the date of diagnosis to death. Recurrence‐free survival (RFS) was defined as the number of months from the date of diagnosis to the event of recurrence [23]. Kaplan–Meier method was used to produce OS and RFS curves in each subgroup of each cohort. Log‐rank test was used to compare the OS and RFS between each subgroup. Univariate and multivariate Cox regression models were used to evaluate independent prognostic factors associated with the survival of patients with HNSCC. Results of Cox regression analyses were reported as hazard ratios (HRs), 95% confidence intervals (95% CIs), and *P*‐values. *P* < 0.05 was considered statistically significant. r software package (http://www.r‐project.org) was used for all statistical analyses.

### 2.8 Cell line cultures

HNSCC cell lines CAL27 was purchased from the American Type Culture Collection (ATCC); HSC3 was obtained from the Japanese Cancer Resources Bank (JCRB); and YD38 was purchased from the Korean Cell Line Bank (KCLB). All cell lines were each purchased within the last 5 years and the identities of some cells were confirmed by short tandem repeat (STR) profiling by KCLB. All cells have a large number of cells in stock after direct purchase. And the cell stock was used within 6 months after freezing and then discarded. HSC3 and YD38 cells were cultured in RPMI supplemented with 10% FBS and 1% penicillin–streptomycin (PS). CAL27 cells were cultured in Dulbecco's modified Eagle's medium (DMEM) supplemented with 10% FBS and 1% PS. The cultures were incubated in an incubator with 5% CO2, at 37 °C.

### 2.9 siRNA constructs and transfection

Synthetic siRNAs specific for *GFP*, *FAT1 #1*, and *FAT1 #2* were purchased from Bioneer (Korea), and nonspecific green fluorescent protein (GFP), 5′‐GCAUCAAGGUGAACUUCAA‐3′ (sense), 5′‐UUGAAGUUCACCUUGAUGC‐3′ (antisense); FAT1 #1, 5′‐CAGUCUUUGCAACCGAUCA‐3′ (sense), 5′‐UGAUCGGUUGCAAAGACUG‐3′ (antisense); FAT1 #2, 5′‐CACUCUUUGAACAACAGAU‐3′ (sense), 5′‐AUCUGUUGUUCAAAGAGUG‐3′ (antisense). Cells were transfected with siRNA using Lipofectamine^®^ RNAiMAX Reagent (Life Technologies, Carlsbad, CA, USA), according to the manufacturer's recommendations.

### 2.10 Clonogenic survival assays

Exponentially growing cells were transfected with GFP siRNA or gene‐specific siRNAs for 24 h and then plated into 6‐well plates. After 24 h, the cells were irradiated with 0, 2, 4, or 8 Gy. Cells were allowed to grow for 10–14 days, followed by fixing and staining with crystal violet solution (0.5% crystal violet in 50% methanol). Each experiment was carried out in triplicate, and colonies containing more than 50 cells were counted. To determine the survival fraction, colony‐forming efficiency was determined, averaged, and normalized to that of the nonirradiated control. Ionizing radiation‐dependent cell survival curves were fitted using a linear quadratic model. The significance of the difference between dose responses was calculated using a two‐way ANOVA test.

### 2.11 Western blot

After irradiation of siGFP‐, siFAT1 #1‐, or siFAT1 #2‐transfected cells, the cells were lysed in RIPA buffer (50 mm Tris/HCl, 150 mm NaCl, 2 mm EDTA, and 1% Triton™ X‐100) containing protease inhibitors (Roche, Mannheim, Germany) and phosphatase inhibitors (Sigma‐Aldrich, Burlington, MA, USA) for 10 min on ice. Equal amounts of samples were separated on 8%–15% SDS/polyacrylamide gel, electrophoresed, and transferred to polyvinylidene difluoride membranes (Millipore, Billerica, MA, USA). The membranes were blocked and probed with primary antibodies against anti‐FAT1 (Bethyl Laboratories Inc., Montgomery, TX, USA), anti‐γ‐H2AX (S139) (Abcam, Cambridge, MA, USA), or anti‐β‐actin (Santa Cruz Biotechnology, Santa Cruz, CA, USA) and incubated with horseradish peroxidase‐conjugated secondary antibodies (Cell Signaling Technology, Danvers, MA, USA).

### 2.12 γ‐H2AX immunofluorescence staining

DNA double‐strand break kinetics were studied using γ‐H2AX foci immunofluorescence staining. The cells were plated on glass coverslips in a 24‐well plate, washed with PBS, and fixed using 10% formalin for 10 min at room temperature, after exposure treatment of siGFP‐, siFAT1 #1‐, or siFAT1 #2‐transfected cells. Following that, the specimens were blocked with the supernatant of 5% BSA/PBS, stirred for 1 h, and incubated with a rabbit‐polyclonal antibody against γ‐H2AX (phospho S139, Abcam) overnight at 4 °C, followed by washing and incubation with Alexa Flour™ 647‐labeled anti‐rabbit secondary antibodies (Abcam) for 1 h at room temperature. The specimens were counter‐stained with 4′,6‐diamidino‐2‐phenylindole (DAPI) and washed in 0.1% PBS‐Tween‐20 before mounting with Vectashield^®^ mounting medium (Vector Laboratories Inc., Burlingame, CA, USA). Fluorescent images were obtained using a confocal microscope (Zeiss LSM710, Jena, Germany).

### 2.13 Detection of cell death via flow cytometry

The cell death rate was analyzed using an Annexin V‐FITC Apoptosis Detection Kit (BioBud, Seongnam, Korea). After exposure treatment to siGFP‐, siFAT1 #1‐, or siFAT1 #2‐transfected cells, the cells were washed twice with ice‐cold PBS. The cells (1 × 10^6^) were then resuspended in 195 μL of binding buffer, and 5 μL of Annexin V‐FITC and propidium iodide (PI) were added to the cell suspension, which was then covered in aluminum foil and incubated at room temperature for 20 min. After incubation, cell death was analyzed using flow cytometry.

### 2.14 Droplet digital PCR copy number assay

The cDNA from tumor tissue of eight patients was analyzed for FAT1 and 23 FAT1 signature genes using ddPCR. The sequences of the primers used in this study are listed in Table S1. ddPCR was performed using a QX200 Droplet Digital PCR (ddPCR) system (Bio‐Rad, Hercules, CA, USA). The final volume of the PCR mixture was 20 µL, containing 10 µL of 2× ddPCR™ Supermix for EvaGreen (Bio‐Rad), 10 pmol of each primer, template DNA, and RNase‐/DNase‐free water. The following PCR conditions were used for ddPCR: 95 °C for 5 min; followed by 40 cycles of 95 °C for 30 s and 60 °C for 1 min (ramping rate reduced to 2 °C·s^−1^); and three final steps at 4 °C for 5 min, 90 °C for 5 min, and a 4 °C indefinite hold to enhance dye stabilization. PCR products were then analyzed using the QX200™ droplet reader and quantasoft™ analysis Software (Bio‐Rad).

### 2.15 Research ethics

This study was conducted in accordance with the tenets of the Declaration of Helsinki and approved by the Institutional Review Board (IRB) of KHUMC prior to its initiation (IRB No. 2018‐05‐046), and all subjects provided written informed consent before being included in this study.

## 3 Results

### 3.1 Development of FAT1‐related gene signature

The alteration frequency of FAT1 is best observed in HNSCC among the various cancers (27.53%; Fig. S1). FAT1‐mutated patients showed lower FAT1 mRNA expression rates than FAT1 wild‐type patients in the TCGA HNSCC cohort (*P* = 4.975e−05, Fig. S2A). Receiver operating characteristic curve analysis of the expression level of FAT1 was performed to distinguish between FAT1 wild‐type and mutated patients, which yielded an area under the curve (AUC) of 0.659 (95% CI = 0.606–0.712, 85.6% sensitivity, and 44.3% specificity at the Youden‐index threshold; Fig. S2B). There were no significant differences in five‐year OS and RFS rates between FAT1 low‐ and high‐expression subgroups, based on the Youden‐index threshold in the TCGA HNSCC cohort (*P* = 0.8 and 0.4, respectively; Fig. S2C,D). In addition, there were no significant differences in five‐year OS and RFS rates between FAT1 wild‐type and mutated subgroups in the TCGA HNSCC cohort (*P* = 0.2 and 0.2, respectively; Fig. S2E,F).

Thus, to develop signatures related to FAT1 mutation and mRNA expression simultaneously, we determined overlapping genes whose expression is correlated with FAT1 mRNA expression and which are differentially expressed between the FAT1 wild‐type and mutated subgroups in the TCGA HNSCC cohort (Fig. S3A). The expression of the 23 genes was tightly associated with mRNA expression and mutation of FAT1 and was selected as the FAT1 signature (Table S2). The FAT1 signature was used to construct the prediction model (Fig. S3B).

**Fig. 1:**
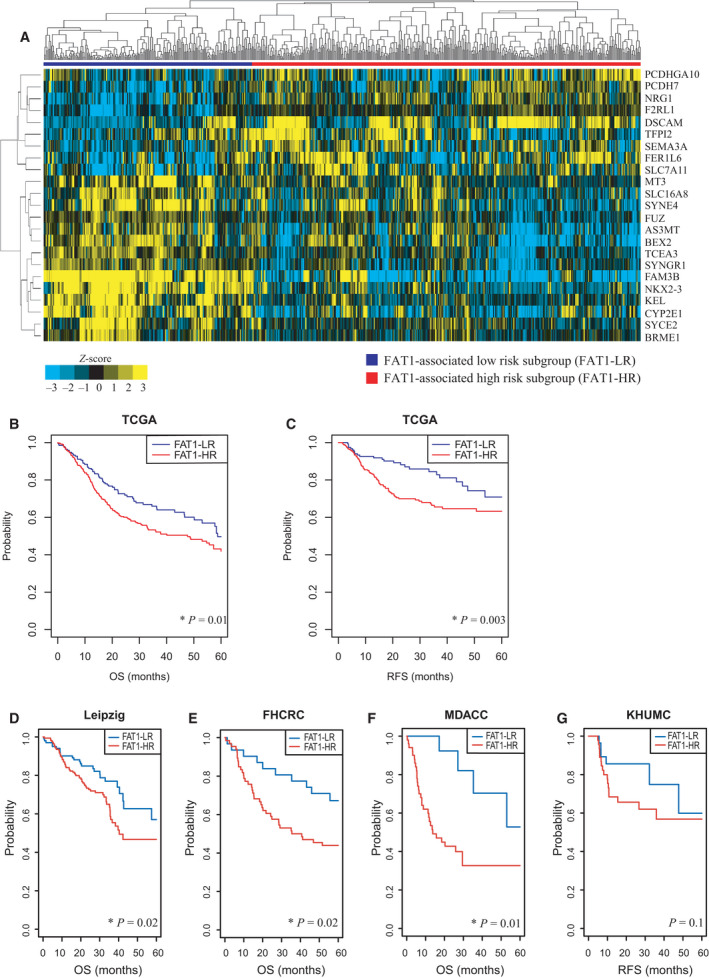
Stratification of HNSCC patients in the TCGA cohort and validation in four independent cohorts, according to the 23 FAT1‐associated signature genes. (A) The TCGA HNSCC patients were classified into FAT1‐LR (*n* = 195) and FAT1‐HR (*n* = 371) subgroups by performing hierarchical clustering. The mRNA expression level in each FAT1‐associated signature gene was converted into a z‐score and shown in a heatmap with row normalized values. (B, C) Five‐year OS and RFS rates of each group were determined using Kaplan–Meier plots. The FAT1‐HR subgroup showed significantly lower five‐year OS and RFS rates than the FAT1‐LR subgroup (*P* = 0.01 and 0.003, respectively). (D‐F) Predicted outcomes in the four independent HNSCC cohorts using FAT1 signature were also depicted using Kaplan–Meier plots. Five‐year OS rates of each group were determined in the Leipzig (*n* = 270), FHCRC (*n* = 97), and MDACC cohorts (*n* = 74; *P* = 0.02, 0.02, and 0.01, respectively). (G) Also, five‐year RFS rates of each group were determined in the KHUMC cohort (*n* = 72, *P* = 0.1). Log‐rank test was used to estimate the *P* value. *P < 0.05. HNSCC, head and neck squamous cell carcinoma; FAT1‐HR, FAT1‐associated high risk; FAT1‐LR, FAT1‐associated low risk; OS, overall survival; RFS, recurrence‐free survival.

Using the FAT1 signature, we performed a hierarchical clustering analysis with the centered correlation coefficient as a measure of similarity and the complete linkage clustering method. Patients in the training dataset (TCGA cohort, *n* = 566) were classified into FAT1‐LR (*n* = 195) and FAT1‐HR (*n* = 371) subgroups, according to the FAT1 signature (Fig. 1A). The rates of FAT1 mutation were significantly higher in the FAT1‐HR subgroup than in the FAT1‐LR subgroup (26.29% vs. 12.75%, *P* = 0.0013). In addition, FAT1 mRNA expression was significantly higher in the FAT1‐HR subgroup than in the FAT1‐LR subgroup (12.50 ± 1.41 vs. 11.79 ± 1.31, *P* = 1.112e−08). The results of Kaplan–Meier analysis and log‐rank test showed that five‐year OS and RFS rates were significantly lower in the FAT1‐HR subgroup than in the FAT1‐LR subgroup, in the TCGA cohort (*P* = 0.01 and 0.03, respectively; Fig. 1B,C).

### 3.2 Independent validation of FAT1‐related gene signature

Twenty‐three FAT1 signatures were validated using four independent cohorts: The Leipzig (*n* = 270), FHCRC (*n* = 97), and MDACC (*n* = 74) cohorts from the National Center for Biotechnology Information Gene Expression Omnibus database, and the KHUMC cohort (*n* = 72) from one tertiary medical center. The details of the clinical and pathological characteristics of each cohort used in this study are shown in Table 1. The FAT1 signature efficiently classified each of the four independent cohorts into FAT1‐LR and FAT1‐HR subgroups, according to the BCCP classifier, which was consistent with the results obtained for the TCGA training dataset. Classification of patients in each test dataset according to FAT1 signature showed worse prognosis in the FAT1‐HR subgroups than in the FAT1‐LR subgroups (Fig. 1D–G). Five‐year OS rates were significantly lower in the FAT1‐HR subgroups than in the FAT1‐LR subgroups, in the Leipzig, FHCRC, and MDACC cohorts (*P* = 0.02, 0.02, and 0.01, respectively; Fig. 1D–F). In addition, five‐year RFS rates tended to be lower in the FAT1‐HR subgroup than in the FAT1‐LR subgroup, in the KHUMC cohort, but the differences were not statistically significant (*P* = 0.1; Fig. 1G). These results supported the prognostic value of the FAT1 signature in the analyzed cohorts.

### 3.3 FAT1 signature as an independent prognostic factor of HNSCC

To assess the independent prognostic factors of HNSCC patients, we performed univariate and multivariate Cox proportional hazards models using patient demographics, social history, and clinical staging, and FAT1 signature of patients in a total of five cohorts (*n* = 1079). FAT1 signature (FAT1‐HR vs. FAT1‐LR subgroup), age (> 60 years vs. ≤ 60 years), and advanced T stage (T3 and T4 vs. T1 and T2) were independent prognostic factors of OS in patients with HNSCC (*P* = 0.025, 0.0494, and 0.0264, respectively; Table S3). FAT1 signature (FAT1‐HR vs. FAT1‐LR subgroup) was also independent prognostic factor of OS in the HPV (−) patients in the five independent HNSCC cohorts (*P* = 0.03115; Table S4). In addition, FAT1 signature and alcohol history (yes vs. no) were independent prognostic factors of RFS in patients with HNSCC (*P* = 0.0094 and 0.0002, respectively; Table S5). Only the FAT1 signature was significantly associated with OS and RFS in patients with HNSCC.

### 3.4 Association of FAT1 signature with HPV status of HNSCC

To assess the association of the FAT1 signature with clinical and pathological features in HNSCC, we compared the FAT1‐LR and FAT1‐HR subgroups in the five HNSCC cohorts (*n* = 1079). Tumor sites were significantly different between the FAT1‐LR and FAT1‐HR subgroups (oral cavity 44.47% vs. 67.47%; oropharynx 31.54% vs. 11.51%; *P* = 2.2e−16; Table S6). In addition, the HPV‐positive ratio was significantly higher in the FAT1‐LR subgroup than in the FAT1‐HR subgroup (36.21% vs. 7.09%, *P* = 4.885e−15).

**Fig. 2:**
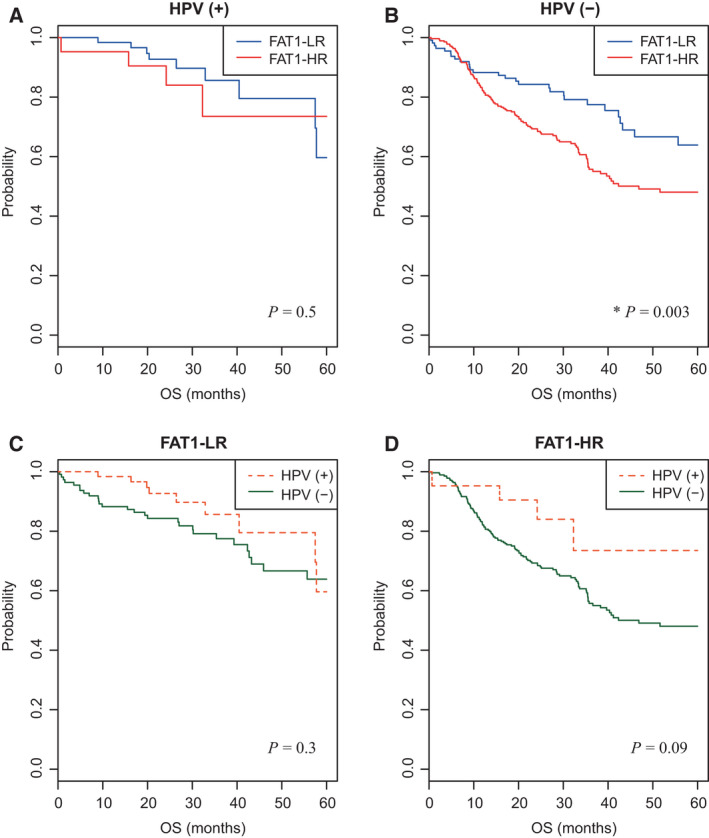
Association of FAT1 signature with HPV in HNSCC patients. (A, B) Five‐year OS rates of FAT1‐LR and FAT1‐HR subgroups in HPV (+) (*n* = 84) and HPV (−) HNSCC patients (*n* = 386) were depicted using Kaplan–Meier plots. (C, D) In the same way, five‐year OS rates of patients were depicted according to HPV in the FAT1‐LR (*n* = 174) and FAT1‐HR subgroups (*n* = 296). The FAT1‐HR subgroup showed significantly lower five‐year OS rates than the FAT1‐LR subgroup, in HPV (‐) HNSCC patients (*P* = 0.003, B). Log‐rank test was used to estimate the *P* value. *P < 0.05. HNSCC, head and neck squamous cell carcinoma; FAT1‐HR, FAT1‐associated high risk; FAT1‐LR, FAT1‐associated low risk; OS, overall survival; RFS, recurrence‐free survival.

HPV status was examined in 470 patients from five HNSCC cohorts. There were no significant differences in five‐year OS rates between the FAT1‐LR and FAT1‐HR subgroups among HPV (+) HNSCC patients (Fig. 2A). However, the FAT1‐HR subgroup showed a significantly lower five‐year OS rate than the FAT1‐LR subgroup among HPV (−) HNSCC patients (*P* = 0.003; Fig. 2B). There were no significant differences in five‐year OS rates between the HPV (+) and HPV (−) subgroups in FAT1‐LR and FAT1‐HR patients (Fig. 2C,D).

### 3.5 Association of FAT1 signature with the result of radiotherapy

**Fig. 3:**
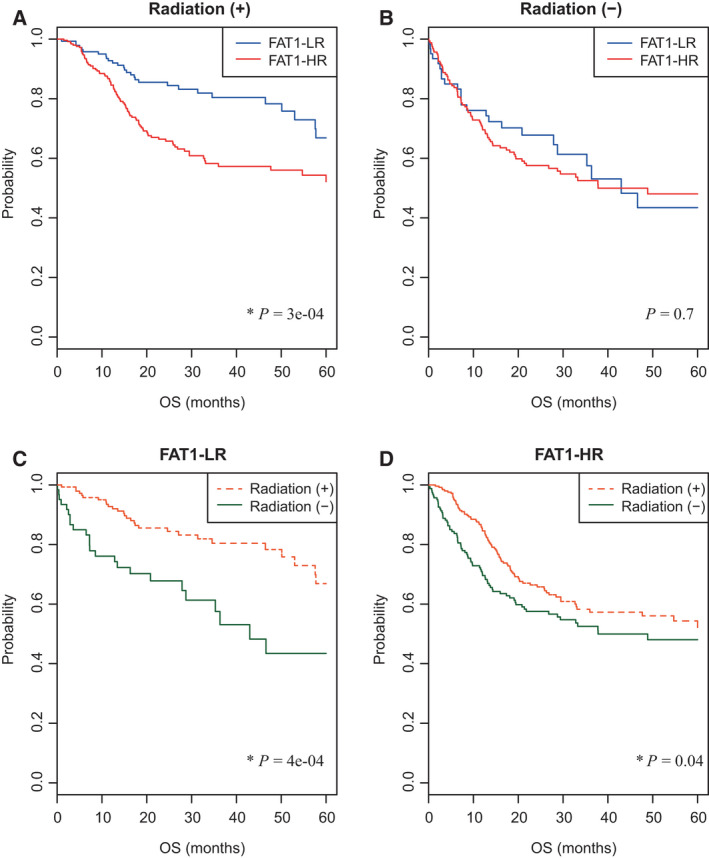
Association of FAT1 signature with radiotherapy in HNSCC patients. (A, B) Five‐year OS rates of FAT1‐LR and FAT1‐HR subgroups in HNSCC patients who did/did not receive radiotherapy (*n* = 391 and 223, respectively) were depicted using Kaplan–Meier plots. (C, D) In the same way, five‐year OS rates of patients were depicted according to radiotherapy in the FAT1‐LR (*n* = 203) and FAT1‐HR subgroups (*n* = 411). Patients in the FAT1‐LR subgroup benefited significantly from radiotherapy (*p* = 3e−04, 4e−04, respectively; A, C). Log‐rank test was used to estimate the *P* value. *P < 0.05. HNSCC, head and neck squamous cell carcinoma; FAT1‐HR, FAT1‐associated high risk; FAT1‐LR, FAT1‐associated low risk; OS, overall survival; RFS, recurrence‐free survival.

The FAT1‐HR subgroup showed significantly lower five‐year OS rates than the FAT1‐LR subgroup, among HNSCC patients who received radiotherapy (*p* = 3e−04; Fig. 3A). However, this difference in five‐year OS rates was not observed among patients who did not receive radiotherapy (Fig. 3B). Similarly, the prognosis was better in patients who received radiotherapy than in those who did not receive radiotherapy, among the FAT1‐LR subgroup (*p* = 4e−04; Fig. 3C). This phenomenon was also observed in the FAT1‐HR subgroup (*P* = 0.04; Fig. 3D). To determine any correlation between the FAT1 signature and radiotherapy in HNSCC, we performed an interaction test for OS. The results of the interaction test showed a significant correlation between the FAT1 signature and radiotherapy (*P* = 0.015). We also performed univariate and multivariate Cox proportional hazards models to assess whether the FAT1 signature was an independent prognostic factor in HNSCC patients treated with radiotherapy. Among various factors including sex, age, social history, clinical staging, and FAT1 signature, FAT1 signature was the only independent prognostic factor in HNSCC patients treated with radiotherapy (HR [95% CI], 1.959 [1.242–3.092]; *P* = 0.0039).

### 3.6 Association of FAT1 signature with the clinical stage of HNSCC patients

**Fig. 4:**
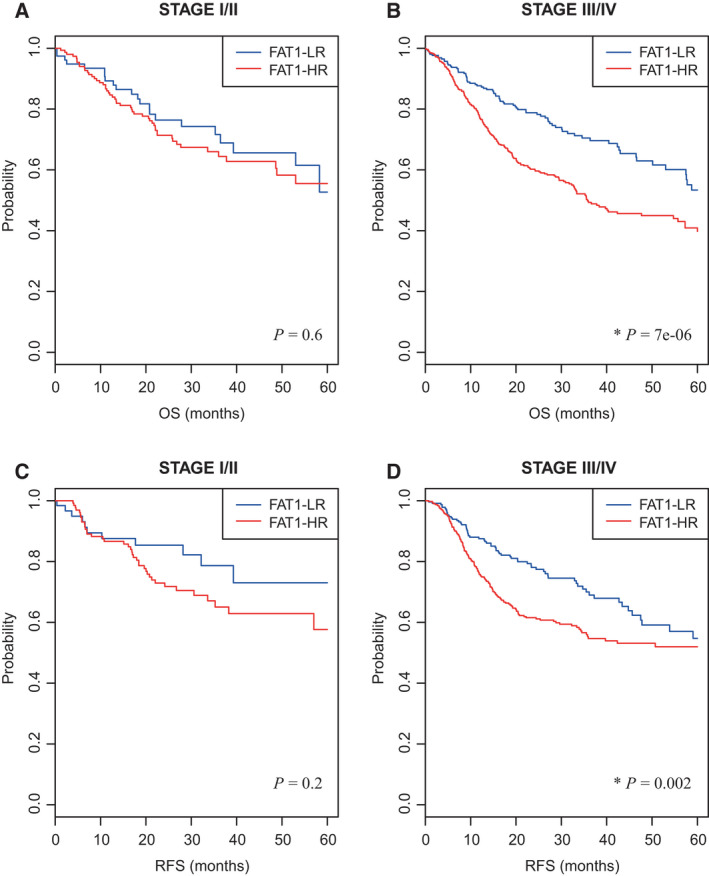
Association of FAT1 signature with clinical stage of HNSCC patients. (A, B) Five‐year OS rates of FAT1‐LR and FAT1‐HR subgroups in HNSCC patients in early‐ and advanced‐stage (Stages I/II and III/IV; *n* = 232 and 714, respectively) were depicted using Kaplan–Meier plots. (C, D) In the same way, five‐year RFS rates of each subgroup were depicted in each clinical stage radiotherapy (*n* = 197 and 645, respectively). The FAT1‐HR subgroup showed significantly lower five‐year OS and RFS rates than the FAT1‐LR subgroup in HNSCC patients with advanced clinical stage (*p* = 7e−06 and 0.002, respectively; B, D). In early‐stage HNSCC patients, there were no significant differences in five‐year OS and RFS rates between the FAT1‐LR and FAT1‐HR subgroups. Log‐rank test was used to estimate the *P* value. *P < 0.05. HNSCC, head and neck squamous cell carcinoma; FAT1‐HR, FAT1‐associated high risk; FAT1‐LR, FAT1‐associated low risk; OS, overall survival; RFS, recurrence‐free survival.

There was no significant difference in the clinical stage (stage I/II vs. stage III/IV) between the FAT1‐LR and FAT1‐HR subgroups (Table S2). We analyzed the prognosis of the FAT1‐LR and FAT1‐HR subgroups in early (I/II)‐ and advanced (III/IV)‐stage HNSCC (Fig. 4). There were no significant differences in prognosis between the FAT1‐LR and FAT1‐HR subgroups in early‐stage HNSCC patients (Fig. 4A,C). However, the FAT1‐HR subgroup showed significantly lower five‐year OS and RFS rates than the FAT1‐LR subgroup, in advanced‐stage HNSCC patients (*p* = 7e−06 and 0.002, respectively; Fig. 4B,D).

### 3.7 Confirmation of association between FAT1 signature and radio‐sensitivity

**Fig. 5:**
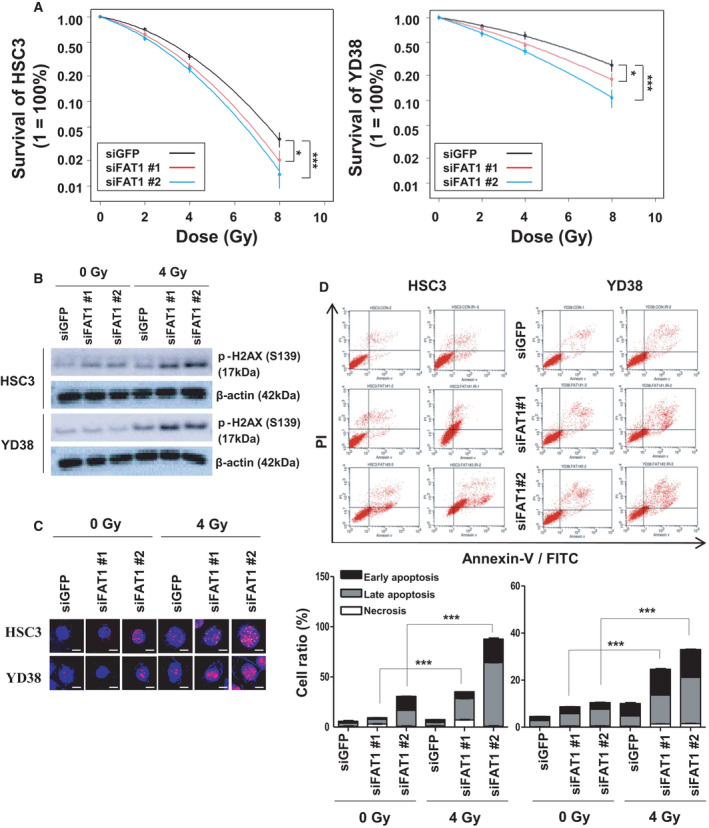
FAT1 is a crucial factor that regulates the sensitivity of radiotherapy in HNSCC. (A) The radiation susceptibility was estimated using a CFA. Colony formation occurred in the siGFP‐, siFAT1 #1‐, or siFAT1 #2‐transfected HSC3 (left) and YD38 (right) cells under radiation doses of 0, 2, 4, and 8 Gy. All experiments were performed in triplicate. The two‐way ANOVA test was used to estimate the *P* value. (HSC3; *P = 0.0193, ***P = 4.77 × 10^−5^ vs. siGFP, YD38; *P = 0.0422, ***P = 0.0003 vs. siGFP). (B) DNA damage to HNSCC cell lines upon radiation exposure was evaluated in terms of level and foci formation of γ‐H2AX (pS139). γ‐H2AX (S139) protein levels in the siGFP‐, siFAT1 #1‐, or siFAT1 #2‐transfected HSC3 and YD38 cells exposed to radiation treatment of 0 or 4 Gy were determined using western blot. β‐Actin was included as an internal loading control. (C) γ‐H2AX foci formation in the same condition was measured using ICC. The cells were stained for γ‐H2AX (green), DNA (blue; DAPI) and observed using confocal microscopy (scale bar: 10 μm). (D) Cell apoptosis was determined by means of Annexin V‐FITC/PI staining using flow cytometry. The siGFP‐, siFAT1 #1‐, or siFAT1 #2‐transfected HSC3 and YD38 cells exposed to radiation treatment of 0 or 4 Gy were analyzed with an Annexin V‐FITC Apoptosis Detection Kit. Cells were classified as healthy (Annexin V^−^, PI^−^), early apoptotic (Annexin V^+^, PI^−^), late apoptotic (Annexin V^+^, PI^+^), and necrotic (Annexin V^−^, PI^+^) (upper) cells. Cell death ratio was calculated as a sum of early apoptosis, late apoptosis, and necrosis percentages. All experiments were performed in triplicate. The t‐test was used to estimate the *P* value. (HSC3 in IR 4 Gy; siFATI1 #1 ***P = 7.37 × 10^−7^, siFAT1 #2 ***P = 2.82 × 10^−6^ vs. IR 0 Gy, YD38 in IR 4 Gy; siFATI1 #1 ***P = 2.29 × 10^−6^, siFAT1 #2 ***P = 9.62 x 10^−7^ vs. IR 0 Gy). All data have been represented as mean ± SD. The number of asterisks denote the level of statistical significance: *P < 0.05 and ***P < 0.001. HNSCC, head and neck squamous cell carcinoma; GFP, green fluorescent protein; PI, propidium iodide; DAPI, 4′,6‐diamidino‐2‐phenylindole.

To further understand the relationship between FAT1 signature and radiotherapy, the genetic information of 15 HNSCC cell lines was obtained using the Cancer Cell Line Encyclopedia, and the cell lines were clustered into FAT1‐LR and FAT1‐HR using the FAT1 signature (Fig. S4A). Among them, HSC3 and YD38 cells were selected as the FAT1‐HR subgroups, and we observed the radiation response in the two cell lines in various ways. First, we confirmed the silencing efficiency of two siRNAs (siFAT1 #1 and #2) in HSC3 and YD38 cells using real‐time quantitative reverse transcriptase PCR analysis (Fig. S4B). Second, to confirm the change in radiation sensitivity due to FAT1 control, a colony‐forming assay (CFA) was performed in HSC3 and YD38 cells under different doses of irradiation. As shown in the clonogenic survival curve, siFAT1 #1‐ or #2‐treated cells were more sensitive to radiation than siGFP‐treated cells, in case of both HSC3 (*P* = 0.0193 and *P* = 4.77 × 10^−5^, respectively) and YD38 (*P* = 0.0422 and *P* = 0.0003, respectively; Fig. 5A). Subsequently, we measured the level of S139 phosphorylated histone H2AX (γ‐H2AX), which is indicative of DNA damage, in HSC3 and YD38 irradiated post‐treatment with siFAT1s or siGFP. When siFAT1s‐ or siGFP‐treated cells were irradiated at 4 Gy, it was identified that the γ‐H2AX level was markedly higher in the siFAT1s‐treated cells than in the siGFP‐treated cells (Fig. 5B). Under the same condition, we confirmed the maintenance of γ‐H2AX foci formation using immunofluorescence staining (Fig. S4C,D); upon counting γ‐H2AX foci in the nucleus, there were significantly higher foci in the siFAT1s‐treated cells, as compared to the siGFP‐treated cells, under 4 Gy irradiation in both the cell lines (Fig. 5C). In addition, to investigate the impact of the regulation of FAT1 on radiation sensitivity in HSC3 and YD38 cells, the cell death rate was examined by combined staining with Annexin V and PI. As shown in Fig. 5D, when HSC3 and YD38 cells were irradiated, the death rates of both siFAT1 #1‐ and FAT1 #2‐treated cells increased approximately three to four times, as compared to that of the nonirradiated cells. In particular, in the group treated with siFAT1 #2+radiation, the cell death rates were 87.7% in HSC3 cells and 33.0% in YD38 cells, in contrast to 7.5% and 10.1%, respectively, in the group treated with siGFP+radiation. As expected from the previous results, it was confirmed that cell death increased exponentially upon irradiation (Fig. 5D).

### 3.8 The crucial role of FAT1 signature for radioresistance in HNSCC

**Fig. 6:**
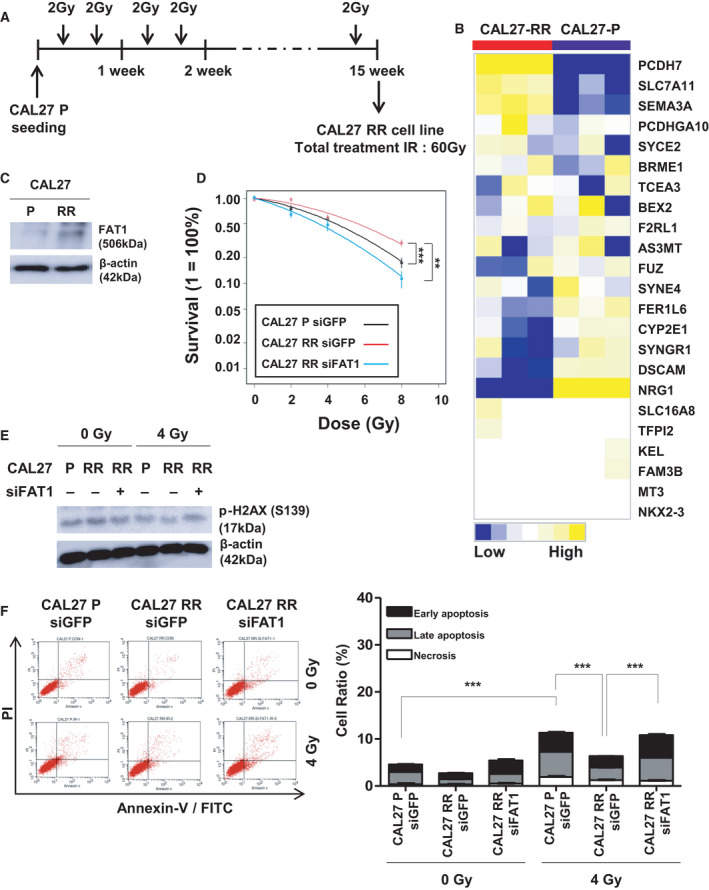
FAT1 regulated sensitivity of radiotherapy in HNSCC. (A) The process of *in vitro* radiation selection in human HNSCC cell line, CAL27. (B) RNA‐sequencing analysis was performed on the established radioresistant cell line, CAL27‐RR (*n* = 3) and parent cell line, CAL27‐P (*n* = 3). Heatmap showing the gene distribution after hierarchical clustering of RNA‐sequencing data. (C) FAT1 protein levels in CAL27‐P and CAL27‐RR cell lines was determined using western blot. β‐Actin was included as an internal loading control. (D) The siGFP‐transfected CAL27‐P, siGFP‐transfected CAL27‐RR, and siFAT1 #2‐transfected CAL27‐RR cells were subjected to a colony‐formation process under radiation doses of 0, 2, 4, 8 Gy. All experiments were performed in triplicate. The two‐way ANOVA test was used to estimate the *P* value. (CAL27 RR siGFP; ***P = 5.45 × 10^−5^ vs. CAL27 P siGFP, **P = 0.0048 vs. CAL27 RR siFAT1). (E) The cells, after radiation exposure, were evaluated for level of foci formation of γ‐H2AX (pS139). The γ‐H2AX (S139) protein levels in CAL27‐P‐siGFP, CAL27‐RR‐siGFP, and CAL27‐RR‐siFAT1 #2 cells, exposed to radiation of 0 or 4 Gy, were determined using western blot. β‐Actin was included as an internal loading control. (F) Cell apoptosis was determined by means of Annexin V‐FITC/PI staining using flow cytometry. CAL27‐P‐siGFP, CAL27‐RR‐siGFP, CAL27‐RR‐siFAT1 #2 cells, treated with radiation of 0 or 4 Gy, were analyzed using an Annexin V‐FITC Apoptosis Detection Kit. Cells were classified as healthy (Annexin V^−^, PI^−^), early apoptotic (Annexin V^+^, PI^−^), late apoptotic (Annexin V^+^, PI^+^), and necrotic (Annexin V^−^, PI^+^) cells (upper). Cell death ratio was calculated as the sum of early apoptosis, late apoptosis, and necrosis percentages. All experiments were performed in triplicate. The t‐test was used to estimate the P value. (CAL27 P siGFP in IR 4 Gy; ***P = 8.69 × 10^−5^ vs. IR 4 Gy, CAL27 RR siGFP in IR 4 Gy; ***P = 1.16 × 10^−4^ vs. CAL27 P siGFP, ***P = 4.81 × 10^−6^ vs. CAL27 RR siFAT1). All data have been presented as mean ± SD. The number of asterisks denote the level of statistical significance: **P < 0.01 and ***P < 0.001. HNSCC, head and neck squamous cell carcinoma; GFP, green fluorescent protein; PI, propidium iodide.

More specifically, we investigated the relationship between FAT1 and resistance to radiotherapy in HNSCC cell lines that acquired radiation resistance. Using the scheme shown in Fig. 6A, we established a highly radioresistant HNSCC cell line, CAL27‐RR, generated from its radiation‐susceptible parental cell line, CAL27‐P, through exposure to 2 Gy radiation twice a week for 15 weeks. We have already confirmed that CAL27‐RR cells have increased resistance to radiation than CAL27‐P cells through over several repeated experiments in the colony formation assay (data not shown). We performed RNA‐sequencing (RNA‐seq) for CAL27‐P and CAL27‐RR cell lines in triplicate, and a hierarchical clustering analysis with the centered correlation coefficient as a measure of similarity and the complete linkage clustering method using the FAT1 signature. These samples were clearly classified into the CAL27‐P (*n* = 3) and CAL27‐RR (*n* = 3) subgroups (Fig. 6B). To determine whether the expression of FAT1 changes when head and neck cancer cells acquire radiation resistance, the expression of FAT1 in the parent cancer cells, CAL27‐P and CAL27‐RR cells were confirmed. As shown in Fig. 6C, we confirmed that FAT1 was upregulated in CAL27‐RR cells, as compared to CAL27‐P cells. Sequentially, we performed CFA using siGFP‐transfected CAL27‐P, siGFP‐transfected CAL27‐RR, and siFAT1‐transfected CAL27‐RR, to identify any change in radioresistance. Prior to the CFA experiment, we confirmed the status of FAT1 protein level in each cell line using western blot (Fig. S5A). As expected, CAL27‐RR cells that acquired radioresistance were less sensitive to radiation than CAL27‐P cells (*P* = 5.453 × 10^−5^). Notably, silencing of FAT1 reversed the resistant phenotypes of CAL27‐RR cells against radiation exposure (*P* = 0.0048, Figs S5B and 6D). In addition, when CAL27‐P, CAL27‐RR, and siFAT1‐transfected CAL27‐RR cells were irradiated with 4 Gy radiation, there was a decrease in the H2AX phosphorylation levels in CAL27‐RR cells, but a conspicuous increase in the FAT1‐silenced CAL27‐RR cells, as compared to CAL27‐P cells (Fig. 6E). In addition, we consistently verified that under irradiation at 4 Gy, the cell death rate also decreased in CAL27‐RR cells, but increased in the FAT1‐silenced CAL27‐RR cells, as compared to that in CAL27‐P cells (Fig. 6F).

### 3.9 The relationship between FAT1 signature and response to radiotherapy in patients with HNSCC

**Fig. 7:**
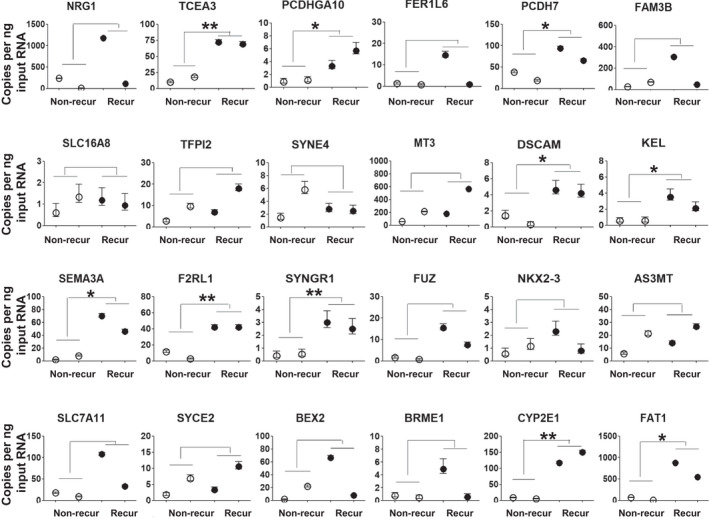
mRNA expression of FAT1‐associated signatures can be used to predict response of radiotherapy in patients with HNSCC. The results of ddPCR‐based expression analysis of 23 genes (NRG1, TCEA3, PCDHGA10, FER1L6, PCDH7, FAM3B, SLC16A8, TFPI2, C19orf46, MT3, DSCAM, KEL, SEMA3A, FRL1, SYNGR1, FUZ, NKX2‐3, AS3MT, SLC7A11, SYCE2, BEX2, C19orf57, and CYP2E1) and FAT1 gene in HNSCC patients with (*n* = 2; recur) and without (*n* = 2; nonrecur) recurrence postradiotherapy. Independent samples *t*‐test was used to compare the values between 2 groups. Poisson error bars are shown for each sample. The number of asterisks denote the level of statistical significance: *P < 0.05 and **P < 0.01. HNSCC, head and neck squamous cell carcinoma; ddPCR, Droplet digital PCR.

Based on the results of the previous experiment, the reliability of FAT1‐associated genes determined using RNA‐seq data was validated by determining the mRNA levels of these genes in HNSCC patients with and without recurrence after radiotherapy. mRNA levels of 23 FAT1‐associated signatures (NRG1, TCEA3, PCDHGA10, FER1L6, PCDH7, FAM3B, SLC16A8, TFPI2, C19orf46, MT3, DSCAM, KEL, SEMA3A, FRL1, SYNGR1, FUZ, NKX2‐3, AS3MT, SLC7A11, SYCE2, BEX2, C19orf57, and CYP2E1), and FAT1 genes were analyzed using ddPCR, which is measured in copies per microliter of input cDNA. We found that FAT1 mRNA expression was significantly higher in HNSCC patients with recurrence than those with nonrecurrence after radiotherapy (*P* = 0.0294; Table S7 and Fig. 7). Interestingly, nine genes (TCEA3, PCDHGA10, PCDH7, DSCAM, F2RL1, SYNGR1, and CYP2E1) out of the FAT1 signature genes were found to have significantly higher expression in the recurrence group than that in the nonrecurrence group, postradiotherapy (*P* = 0.0031, 0.0493, 0.0499, 0.0128, 0.0422, 0.0246, 0.0081, 0.0061, and 0.0081, respectively; Table S7 and Fig. 7).

### 3.10 Pathway analysis

The FAT1 signature genes were submitted to the Database for Annotation, Visualization, and Integrated Discovery (DAVID) bioinformatics resources 6.8 to discover the gene ontology categories with significantly enriched gene numbers. The significance of the gene annotation with a p‐value less than 0.05 was determined with two‐tailed Fisher's exact test. A total of 23 FAT1 signature gene analysis with DAVID bioinformatics resources 6.8 identified one significant REACTOME pathway: R‐HAS‐21099A (Basigin interactions) pathway (*P* = 3.3E−2, related genes = SLC16A8, SLC7A11).

## 4 Discussion

In this study, we developed and validated the FAT1 signature in five independent HNSCC cohorts. We observed that patients in the FAT1‐LR subgroup had a better prognosis than those in the FAT1‐HR subgroup. Similar results were observed in HPV (−) HNSCC patients, but not in HPV (+) HNSCC patients. Furthermore, we observed that the FAT1 signature could accurately predict outcomes in patients receiving radiotherapy. The association between FAT1 signature and radiosensitivity was confirmed using HNSCC cell lines. Thus, the FAT1 signature in HNSCC may have the ability to identify patients with HNSCC who are refractory to radiotherapy and need treatment intensification or personalized treatment.

The relationship between FAT1 and patient prognosis is often interpreted in terms of different aspects, because FAT1 is involved in various signaling pathways, and it might be altered by various mechanisms during tumorigenesis. Kim et al. [16] found that FAT1 mutations were significantly associated with better OS, but the expression level of FAT1 was not associated with OS in HPV (−) HNSCC patients. These results were evaluated from the TCGA cohort and an independent cohort of patients with gingivobuccal squamous cell carcinoma from the International Cancer Genome Consortium (ICGC) portal. Recently, Lin et al. [14] found that FAT1 mutation and downregulation resulted in higher tumor progression and recurrence in patients with HNSCC. In this study, multiplex PCR‐based next‐generation sequencing was performed in 101 HNSCC patients who received therapeutic surgery. In addition, Hsu et al. [17] found that high FAT1 expression was positively correlated with poor prognosis in patients with oral squamous cell carcinoma in the TCGA cohort, which was contrary to the prognosis of HNSCC patients according to FAT1 mutation and mRNA expression. This may be because of the diverse mutation subtype of FAT1, individual cut‐point to divide low and high FAT1 expression, as well as, the different cohorts used in these studies. In addition, the number of samples in the validation set (ICGC cohort) was small (*n* = 37), because the FAT1 mutation status available from public data was limited. Thus, to validate FAT1 alterations in other cohorts, FAT1 mRNA expression, in addition to FAT1 mutations, need to be considered together.

In this study, we considered FAT1 mutation and mRNA expression simultaneously in the TCGA HNSCC cohort and developed 23 FAT1‐related signatures. The FAT1 signature was validated in the other four cohorts appropriately, although these cohorts had no FAT1 mutation status data. Our results showed that FAT1‐HR (FAT1 mutated and high expression) subgroup had a shorter survival and recurrence‐free period than FAT1‐LR (FAT1 wild‐type and low expression) subgroup. Although our data contradict some of the previously reported results showing poor prognosis in HNSCC patients with wild‐type or low expression of FAT1 [14, 16], our results are noteworthy, since they include a variety of mechanisms that can be regulated by FAT1.

Radiotherapy is an important treatment modality for early‐ and advanced‐stage HNSCC, but tumors respond heterogeneously to this treatment modality. There is a need to identify factors associated with response to radiotherapy in HNSCC patients; thus, several biomarkers have been developed for personalized therapy, such as intensified radiation schedules or other treatment planning [24, 25]. However, the association between FAT1 alteration status and response to radiotherapy in HNSCC has not been studied. Our study showed that the FAT1‐LR subgroup benefited significantly from radiotherapy, as compared to the FAT1‐HR subgroup. YAP1 overexpression plays an important role in conferring radioresistance in several cancers [26, 27]. Because FAT1 inhibits YAP1 by binding to Scribble [28], the FAT1‐HR subgroup, which displays higher FAT1 mutation rates, was expected to show higher resistance to radiotherapy than the FAT1‐LR subgroup.

We also discovered HNSCC cell lines that were very similar to the FAT1‐HR group of HNSCC patients and confirmed that FAT1 gene silencing in FAT1‐HR cell lines increased the radiosensitivity. In addition, we confirmed that FAT1‐HR cell lines can increase radiosensitivity by promoting cell death, by slowing down the repair of DNA damage during radiotherapy, due to the downregulation of FAT1 expression. In particular, this may explain why HNSCC patients with FAT1‐HR have a poor prognosis for radiotherapy. More specifically, we questioned whether there is a correlation between the acquisition of radioresistance and changes in the FAT1 signatures. It was very interesting the expression of FAT1 was strikingly increased in the HNSCC cell line that acquired radioresistance. In addition, three CAL27‐P and three CAL29‐RR cell lines could be clearly distinguished when clustered by the 23 FAT1 signatures. Thus, we concluded that the FAT1 signature can be a clear indicator that allows to distinguish between radioresistance and nonradioresistance in HNSCC patients.

Our study has the following limitations. Our cohort (KHUMC cohort) showed a better prognosis in the FAT1‐LR subgroup than in the FAT1‐HR subgroup, but there were no significant differences between the two subgroups. This might be due to differences in other clinical and pathological characteristics between the cohorts or the relatively small sample size of our cohort. The other four public independent cohorts showed significantly better prognosis in the FAT1‐LR subgroup than in the FAT1‐HR subgroup.

Nevertheless, to the best of our knowledge, this is the first study to assess prognosis in HNSCC patients while considering both FAT1 mutation and mRNA expression together. Because FAT1 functions as a tumor suppressor or promoter, depending on the variety of cancer, FAT1 alteration data need to be analyzed comprehensively, while considering the association between FAT1 and other genes. Thus, we clarified that the changes in FAT1‐related genes, not FAT1 alone, were significantly related to patient prognosis and were closely related to the response of HNSCC patients to radiation therapy. In addition, the Cox proportional hazards model showed that the FAT1 signature was an independent prognostic factor that influences the OS and RFS of HNSCC patients.

## 5 Conclusion

In conclusion, we developed FAT1 signatures that play an important role in predicting the prognosis of patients with HNSCC. Also, FAT1 signature was associated with the response to radiotherapy, advanced stage, and HPV status in HNSCC patients. Therefore, our data provided evidence that FAT1 signature may help design personalized treatments for HNSCC patients who were classified in more detail.

## Conflict of interest

The authors declare no potential conflicts of interest.

### Peer Review

The peer review history for this article is available at https://publons.com/publon/10.1002/1878‐0261.13171.

## Author contributions

SIK: study design, data collection and analysis, writing, revising article, final approval of the version. SRW: study design, data collection and analysis, writing, revising article, final approval of the version. JKN: data collection and analysis, final approval of the version. MKL: data collection and analysis, final approval of the version. YCL: data collection and analysis, revising article, final approval of the version. JWL: data collection and analysis, revising article, final approval of the version. SGK: data collection and analysis, revising article, final approval of the version. YGE: study design, data collection and analysis, revising article, final approval of the version, supervising this study.

## Supporting information


**Fig. S1.** FAT1 alteration in HNSCC.

**Fig. S2.** FAT1 mRNA expression and mutation status in the TCGA HNSCC patients.

**Fig. S3.** Construction of the prediction model.

**Fig. S4.** FAT1 is a crucial factor for maintenance repair of radiotherapy‐induced DNA damage in FAT1‐HR HNSCC cell lines.

**Fig. S5.** FAT1 modulated radiation sensitivity in the radioresistant NHSCC cell line.

**Table S1.** Primers used for the twenty‐three gene signatures associated with FAT1.
**Table S2.** Twenty‐three gene signatures associated with FAT1 mutation and mRNA expression in the TCGA HNSCC cohort.
**Table S3.** Univariate and multivariate analyses of characteristics associated with overall survival in the five independent HNSCC cohorts (n = 1075).
**Table S4.** Univariate and multivariate analyses of characteristics associated with overall survival in the HPV (‐) patients in the five independent HNSCC cohorts (n = 386).
**Table S5.** Univariate and multivariate analyses of characteristics associated with recurrence‐free survival in five independent HNSCC cohorts (n = 872).
**Table S6.** Association of FAT1 signature with clinical and pathological features in five independent HNSCC cohorts (n = 1079).
**Table S7.** Analysis of mRNA expression of the 23 FAT1‐associated signature genes and FAT1 gene in the KHU HNSCC cohort.

**Appendix S1.** Real‐time quantitative reverse transcriptase PCR analysis.

## Data Availability

The data that support the findings of this study are available from the corresponding author upon reasonable request.
