# Supplementary figures and images for: Clinical significance of FAT1 gene mutation and mRNA expression in patients with head and neck squamous cell carcinoma
Source: Mol Oncol. 2022 Jan 13;16(8):1661–79. doi: 10.1002/1878-0261.13171 (PMC9019907; doi:10.1002/1878-0261.13171)

## Supplementary Fig. 1

A

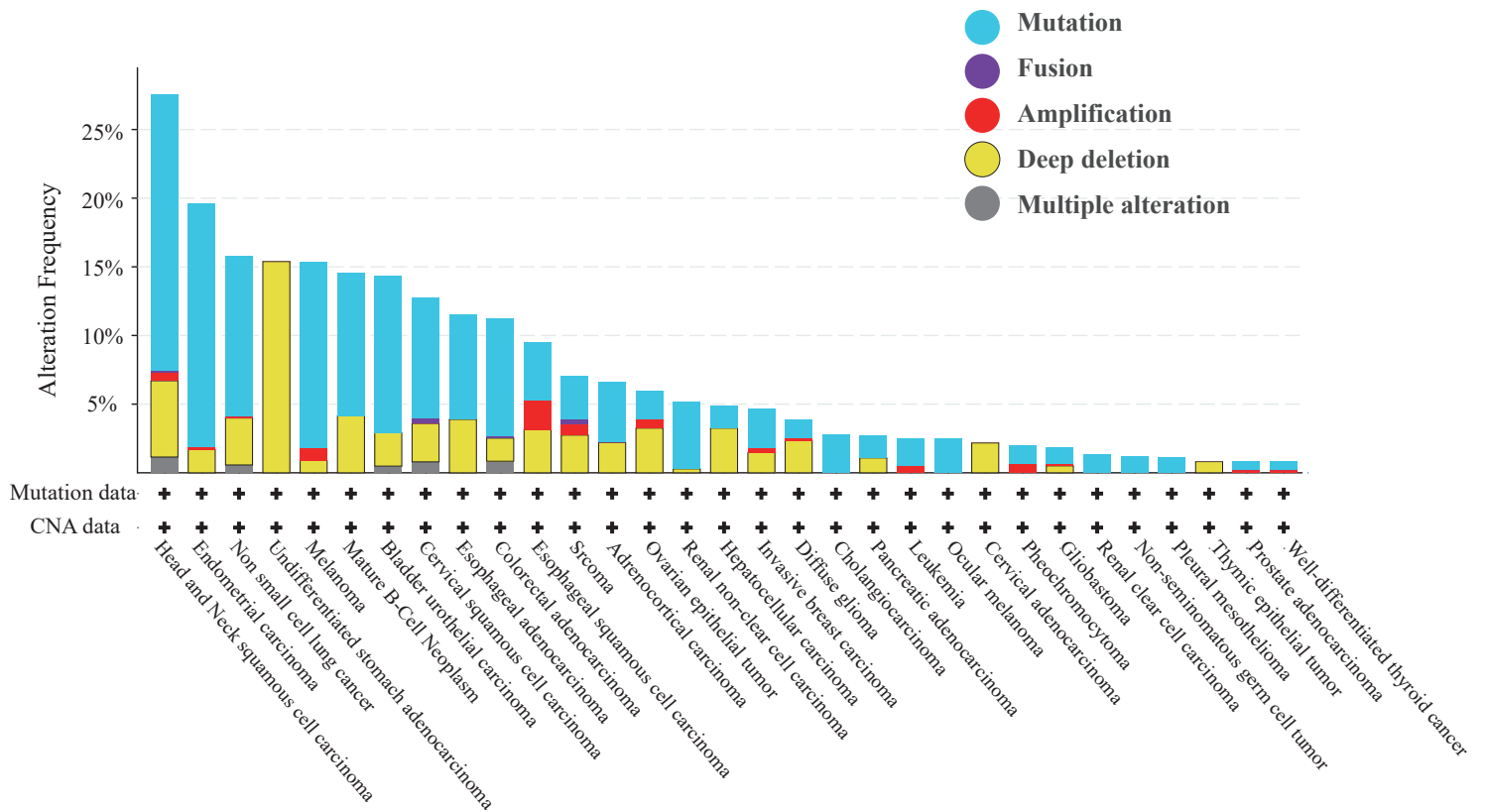

# B

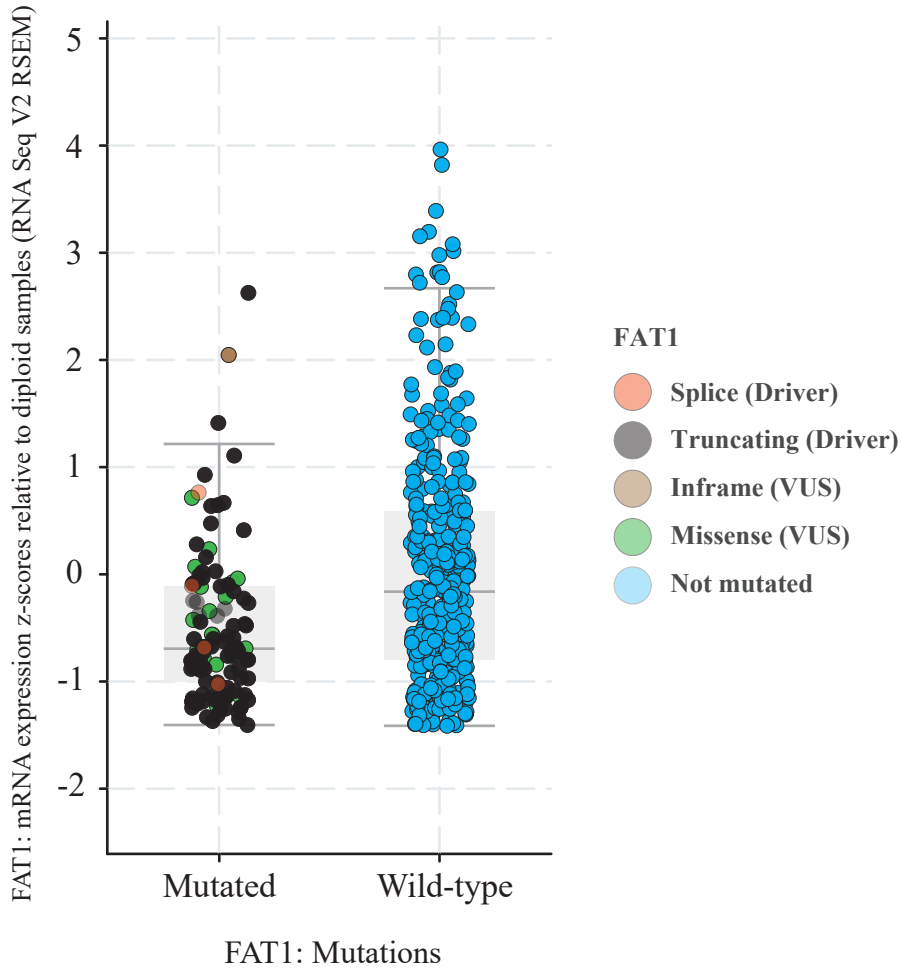

Supplement: Supplementary file 1 — Fig. S1. FAT1 alteration in HNSCC. [file MOL2-16-1661-s002.pdf]

Supplementary Fig. 2

A

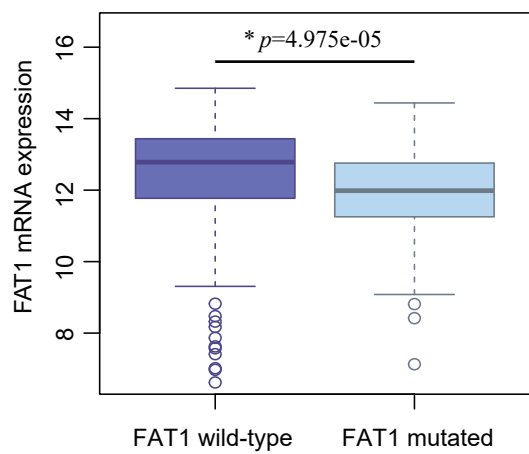

B

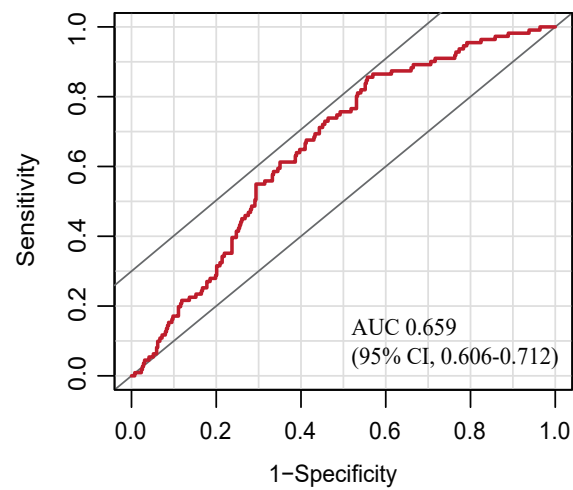

C

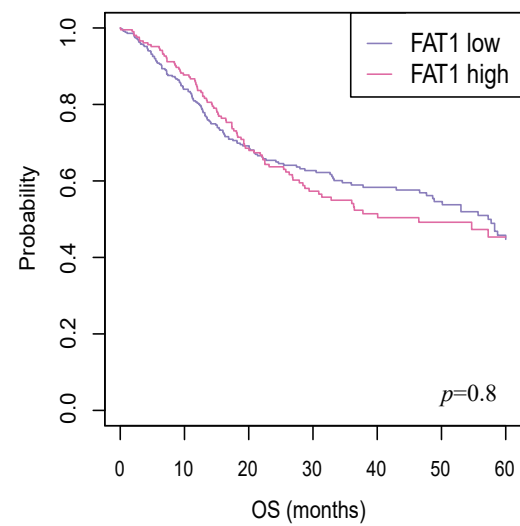

D

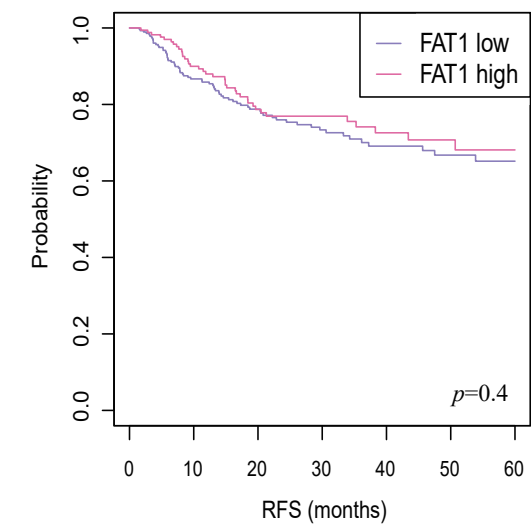

E

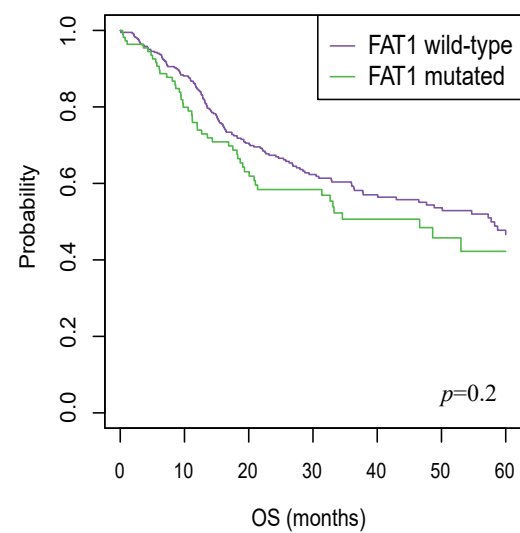

F

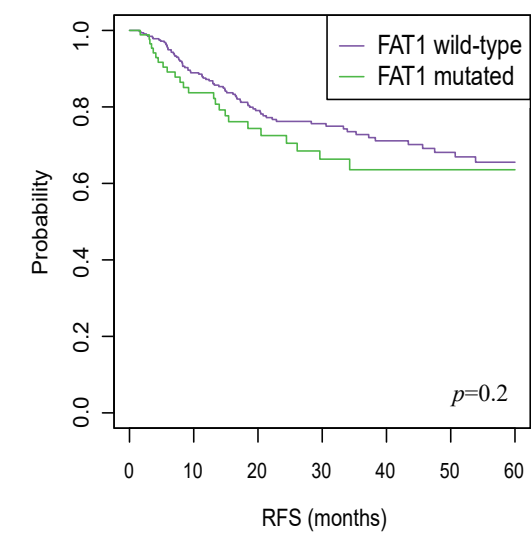

Supplement: Supplementary file 2 — Fig. S2. FAT1 mRNA expression and mutation status in the TCGA HNSCC patients. [file MOL2-16-1661-s005.pdf]

# Supplementary Fig. 3

A

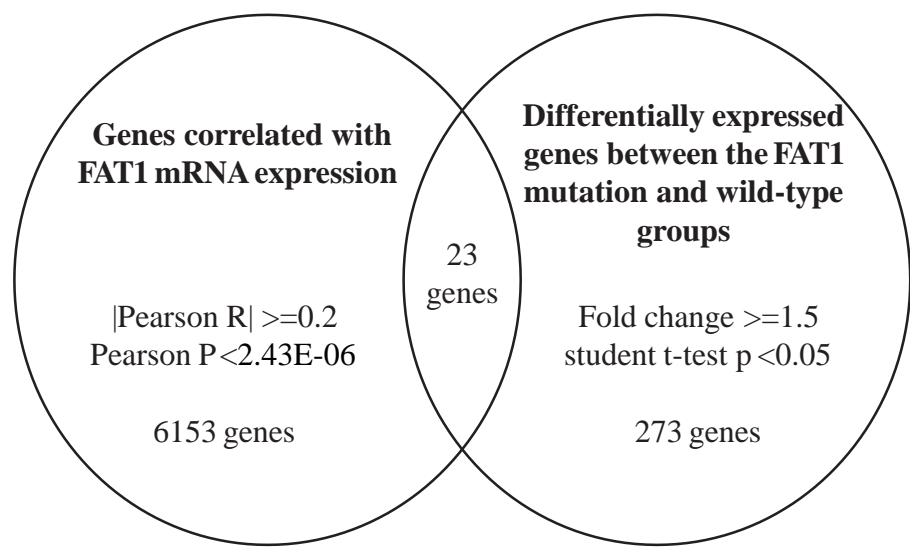

B

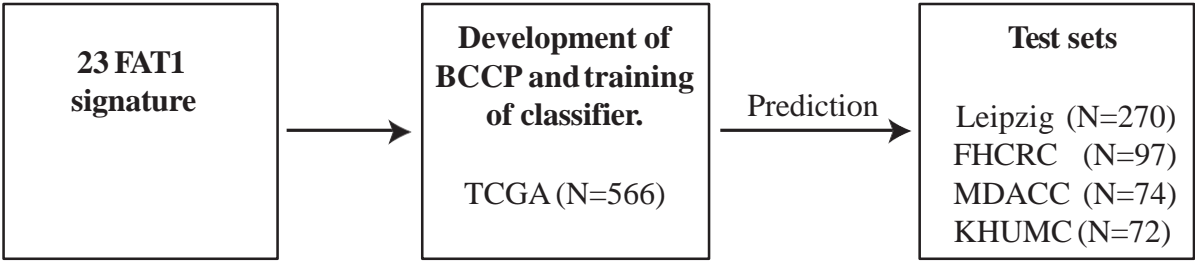

Supplement: Supplementary file 3 — Fig. S3. Construction of the prediction model. [file MOL2-16-1661-s007.pdf]

Supplementary Fig. 4

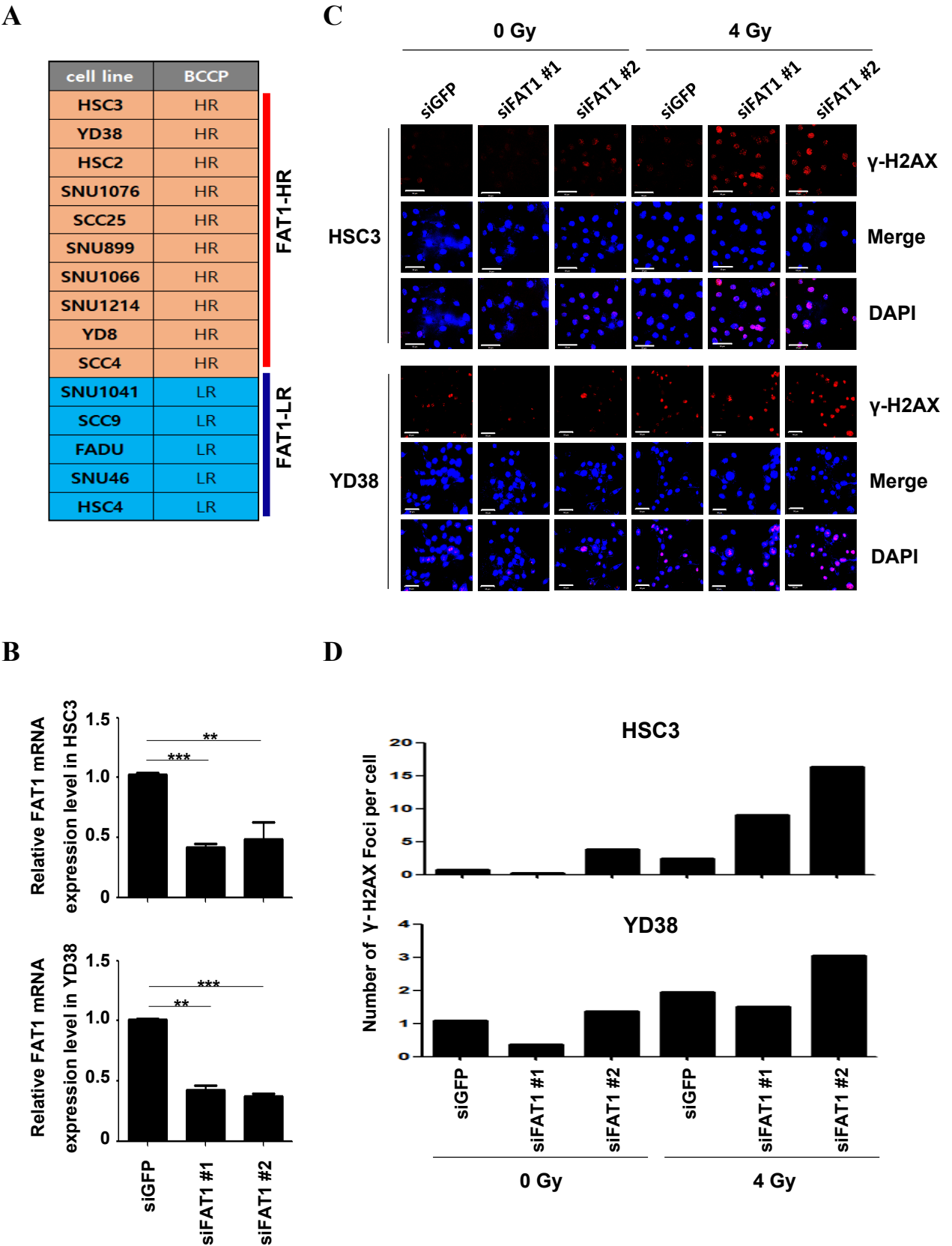

Supplement: Supplementary file 4 — Fig. S4. FAT1 is a crucial factor for maintenance repair of radiotherapy‐induced DNA damage in FAT1‐HR HNSCC cell lines. [file MOL2-16-1661-s001.pdf]
