# Supplementary material for: Clinical significance of FAT1 gene mutation and mRNA expression in patients with head and neck squamous cell carcinoma
Source: Mol Oncol. 2022 Jan 13;16(8):1661–79. doi: 10.1002/1878-0261.13171 (PMC9019907; doi:10.1002/1878-0261.13171)
Supplement: Supplementary file 5 — Fig. S5. FAT1 modulated radiation sensitivity in the radioresistant NHSCC cell line. [file MOL2-16-1661-s003.ppt]

## Slide 1
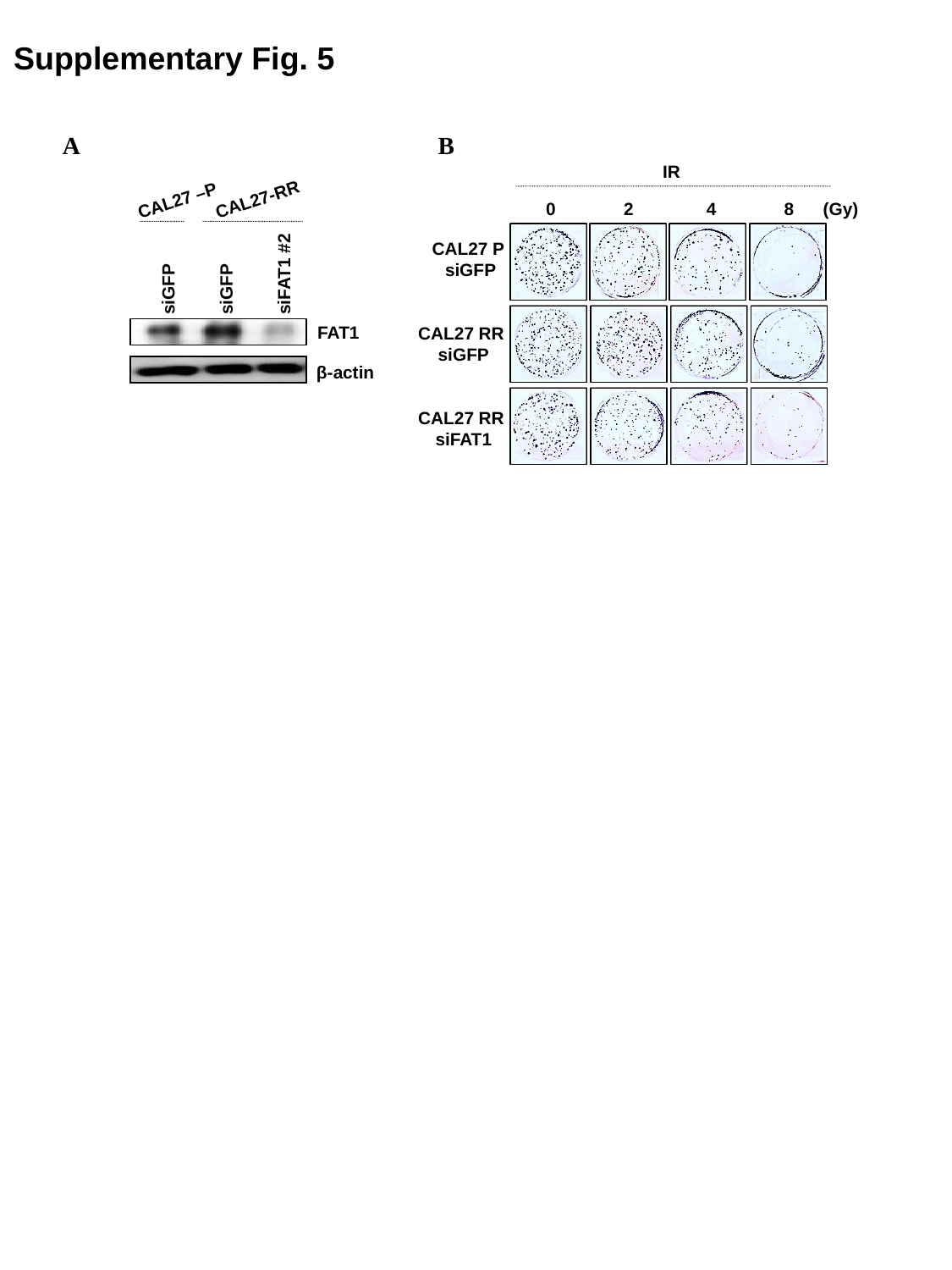

Supplementary Fig. 5
A
B
IR
CAL27-RR
CAL27 –P
0 2 4 8 (Gy)
CAL27 P
siGFP
siFAT1 #2
siGFP
siGFP
FAT1
CAL27 RR
siGFP
β-actin
CAL27 RR
siFAT1
