# Supplementary material for: Clinical significance of FAT1 gene mutation and mRNA expression in patients with head and neck squamous cell carcinoma
Source: Mol Oncol. 2022 Jan 13;16(8):1661–79. doi: 10.1002/1878-0261.13171 (PMC9019907; doi:10.1002/1878-0261.13171)
Supplement: Supplementary file 6 — Table S1. Primers used for the twenty‐three gene signatures associated with FAT1. Table S2. Twenty‐three gene signatures associated with FAT1 mutation and mRNA expression in the TCGA HNSCC cohort. Table S3. Univariate and multivariate analyses of characteristics associated with overall survival in the five independent HNSCC cohorts (n = 1075). Table S4. Univariate and multivariate analyses of characteristics associated with overall survival in the HPV (‐) patients in the five independent HNSCC cohorts (n = 386). Table S5. Univariate and multivariate analyses of characteristics associated with recurrence‐free survival in five independent HNSCC cohorts (n = 872). Table S6. Association of FAT1 signature with clinical and pathological features in five independent HNSCC cohorts (n = 1079). Table S7. Analysis of mRNA expression of the 23 FAT1‐associated signature genes and FAT1 gene in the KHU HNSCC cohort. [file MOL2-16-1661-s008.docx]

**Supplementary Table 1.** Primers used for the twenty-three gene signatures associated with FAT1

| Gene symbol | Forward primer sequences (5′–>3′) | Reverse primer sequences (5′–>3′) |
| --- | --- | --- |
| NRG1  TCEA3  PCDHGA10  FER1L6  PCDH7  FAM3B  SLC16A8  TFPI2  SYNE4  MT3  DSCAM  KEL  SEMA3A  F2RL1  SYNGR1  FUZ  NKX2-3  AS3MT  SLC7A11  SYCE2  BEX2  BRME1  CYP2E1  FAT1 | CAAAGAAGGCAGAGGCAAAG  CCTGAAGGCGGACGATGATT  GGCTTGACAGGTGTGTCCG  CAGTCGGAGCAGTGCCTTTA  AGAGTTGCTATGACAGCGGG  CCATTGGCTGGTGGCCTG  ATGCTAGCCATGCTCTACGG  GCCAACAGGAAATAACGCGG  CCCGAGATGGTCAACACCC  AGACCTGCCCCTGCCCTTC  TCCTTGTTCCAGAGCTTCGC  ACCAAAGTGAGGAAGAGCCG  GCAATTACCTCTGCCATGCG  CAGTGGCACCATCCAAGGAA  CACTGAACGAAGGGACGGAC  GCCCTTGGGGGATAAAGAGC  GAACATGAAGAGGAGCCCGA  GAGGCATATCGGGTGCTGAA  TGCCCAGATATGCATCGTCC  GGCAAAAGGACCATGCACTC  GCAGAAAATGGTGGTTTGCGG  CGAGTTAGGTGGCCAGAACC  GGCTCGCATGGAGTTGTTTC  CCTTCCAACAGCCACATCCACTAC | AACTGGTTTCACACCGAAGG  CAGCAGAACTTCCAGCGA  AGAAACGCCAGTCCGTGT  ACACGTCGACTTTTGCTTGC  CTGCCTCACCAACACTGCC  ACAGGGGTGCATCTGGAATG  GGAAGCTAGGATCATGCCCG  AGAAATTGTTGGCGTTGCCC  GCTCAGCCTCTAGTACCTCCA  TGCTTCTGCCTCAGCTGCC  CCGTGGCTAGGTACCATCTG  GCCAGGTAATGATCCCGGAG  ACCAGACCTTCTGGCTAGGT  TTCCAGTGACGTGGGATGTG  CAATCTGGTACCGCTGGAAGG  GGTTCCTCAGTCCCCAACAC  AAGACCTGGGCTTGCGAGAA  AAAGCACCACCCAGACACTC  TCTTCTTCTGGTACAACTTCCAGT  TTCTGAAGCAGTGGCCTGAG  CTGGGCCTATCCTTGCAGTC  CAGGTTGGAGAGCTCAACGAT  TCATGAGCGGGGAATGACAC  TTGAACCGTGAGCGTGTAACCTG |

**Supplementary Table 2.** Twenty-three gene signatures associated with FAT1 mutation and mRNA expression in the TCGA HNSCC cohort

|  |  | Pearson’s correlation with gene expression of FAT1 in the TCGA HNSCC cohort | | Fold change between FAT1 wild-type and mutated subgroups in the TCGA HNSCC cohort | |
| --- | --- | --- | --- | --- | --- |
| Gene symbol | Gene name | Correlation coefficient | p-value | \|log2(FC)\| | p-value |
| NRG1  TCEA3  PCDHGA10  FER1L6  PCDH7  FAM3B  SLC16A8  TFPI2  SYNE4  MT3  DSCAM  KEL  SEMA3A  F2RL1  SYNGR1  FUZ  NKX2-3  AS3MT  SLC7A11  SYCE2  BEX2  BRME1  CYP2E1 | neuregulin 1  transcription elongation factor A3  protocadherin gamma subfamily A, 10  fer-1-like 6  protocadherin 7  family with sequence similarity 3 member B  solute carrier family 16 member 8  tissue factor pathway inhibitor 2  spectrin repeat-containing nuclear envelope family member 4  metallothionein 3  DS cell adhesion molecule  Kell blood group, metallo-endopeptidase  semaphorin 3A  F2R-like trypsin receptor 1  synaptogyrin 1  fuzzy planar cell polarity protein  NK2 homeobox 3  arsenite methyltransferase  solute carrier family 7 member 11  synaptonemal complex central element protein 2  brain expressed X-linked 2  break repair meiotic recombinase recruitment factor 1  cytochrome P450 family 2 subfamily E member 1 | 0.34358012  -0.314021294  0.299627274  0.298037654  0.287825399  -0.263363759  -0.259669548  0.257741113  -0.248746826  -0.246266504  0.245690381  -0.245469696  0.240863542  0.240765747  -0.236456348  -0.224811096  -0.222251504  -0.221779581  0.213127599  -0.211082984  -0.207422204  -0.202646125  -0.201621073 | 3.95122E-17†  2.0209E-14†  3.31047E-13†  4.46603E-13†  2.92607E-12†  1.95452E-10†  3.55603E-10†  4.84225E-10†  1.97705E-09†  2.88645E-09†  3.14981E-09†  3.25675E-09†  6.48984E-09†  6.58455E-09†  1.239E-08†  6.43748E-08†  9.13958E-08†  9.74521E-08†  3.08153E-07†  4.01717E-07†  6.41586E-07†  1.16703E-06†  1.32448E-06† | 0.586776864  0.739699582  0.619421413  0.597916339  0.728669485  0.989837225  0.807782792  0.691933292  0.612638279  0.652068136  0.645362223  0.633336375  0.634249287  0.643103685  0.63361366  0.636932692  0.935343055  0.685115137  0.610994158  0.691808078  0.839379646  0.692800098  0.587455696 | 0.002037255  4.11302E-06  0.005106866  0.017140061  0.000328648  0.004256853  1.158E-05  0.012657556  0.01038216  0.000348717  0.013412239  0.00503767  0.002293514  4.57104E-05  8.70473E-05  1.4231E-06†  0.000267148  0.000902165  0.005828962  0.000124706  0.000537607  9.11983E-07†  0.012399038 |

TCGA, The Cancer Genome Atlas; HNSCC, head and neck squamous cell carcinoma; FC, fold change

† *p* <2.43E-06

**Supplementary Table 3.** Univariate and multivariate analyses of characteristics associated with overall survival in the five independent HNSCC cohorts (n=1075)

| Characteristics | Univariate | | Multivariate | |
| --- | --- | --- | --- | --- |
|  | HR (95% CI) | p-value | HR (95% CI) | p-value |
| FAT1 signature (FAT1-HR subgroup) | 1.655 (1.330–2.060) | 6.27e-06 * | 1.444 (1.137–1.834) | 0.0025 * |
| Gender (male) | 1.219 (0.661–1.018) | 0.0727 |  |  |
| Age (>60 years) | 1.232 (1.013–1.497) | 0.0362 * | 1.236 (1.001–1.529) | 0.0494 * |
| Alcohol (yes) | 0.863 (0.674–1.105) | 0.242 |  |  |
| Smoking (yes) | 0.953 (0.758–1.199) | 0.683 |  |  |
| Anatomic site (oropharynx) | 0.811 (0.705–0.933) | 0.0034 * | 0.892 (0.767–1.036) | 0.1353 |
| Primary tumor (T3 & T4) | 1.022 (1.012–1.033) | 1.86e-05 * | 1.018 (1.002–1.035) | 0.0264 * |
| Regional lymph node (N+) | 1.207 (0.981–1.485) | 0.0752 |  |  |
| Stage (stage III & IV) | 1.021 (1.010–1.032) | 0.0002 * | 1.000 (0.982–1.019) | 0.9812 |

HNSCC, head and neck squamous cell carcinoma; HR, hazard ratio; CI, confidence interval; FAT1-HR, FAT1-associated high risk

**p*<0.05

**Supplementary Table 4.** Univariate and multivariate analyses of characteristics associated with overall survival in the HPV (-) patients in the five independent HNSCC cohorts (n=386)

| Characteristics | Univariate | | Multivariate | |
| --- | --- | --- | --- | --- |
|  | HR (95% CI) | p-value | HR (95% CI) | p-value |
| FAT1 signature (FAT1-HR subgroup) | 1.8498 (1.223–2.798) | 0.00359 * | 1.7669 (1.0529–2.965) | 0.03115 * |
| Gender (male) | 1.096 (0.7319–1.64) | 0.658 |  |  |
| Age (>60 years) | 0.9006 (0.6469–1.254) | 0.535 |  |  |
| Alcohol (yes) | 1.695 (0.8523–3.372) | 0.132 |  |  |
| Smoking (yes) | 0.8410 (0.4835–1.463) | 0.54 |  |  |
| Anatomic site (oropharynx) | 0.8791 (0.6938–1.114) | 0.286 |  |  |
| Primary tumor (T3 & T4) | 1.042 (1.02–1.066) | 0.00022 * | 1.0251 (0.9959–1.055) | 0.09210 |
| Regional lymph node (N+) | 1.5800 (1.281–1.949) | 1.95e-05 * | 1.4314 (1.1310–1.812) | 0.00284 * |
| Stage (stage III & IV) | 1.05360 (1.031-1.077) | 2.06e-06 * | 1.0087 (0.9671–1.052) | 0.68697 |

HNSCC, head and neck squamous cell carcinoma; HR, hazard ratio; CI, confidence interval; FAT1-HR, FAT1-associated high risk

**p*<0.05

**Supplementary Table 5.** Univariate and multivariate analyses of characteristics associated with recurrence-free survival in five independent HNSCC cohorts (n=872)

| Characteristics | Univariate | | Multivariate | |
| --- | --- | --- | --- | --- |
|  | HR (95% CI) | p-value | HR (95% CI) | p-value |
| FAT1 signature (FAT1-HR subgroup) | 1.496 (1.151–1.944) | 0.0026 * | 1.469 (1.099–1.964) | 0.0094 * |
| Gender (male) | 1.247 (0.922–1.686) | 0.153 |  |  |
| Age (>60 years) | 1.113 (0.878–1.411) | 0.378 |  |  |
| Alcohol (yes) | 1.987 (1.375–2.873) | 0.0003 * | 2.035 (1.399–2.959) | 0.0002 * |
| Smoking (yes) | 1.038 (0.808–1.334) | 0.769 |  |  |
| Anatomic site (oropharynx) | 1.097 (0.958–1.255) | 0.179 |  |  |
| Primary tumor (T3 & T4) | 1.018 (1.006–1.029) | 0.0028 * | 1.017 (0.999–1.035) | 0.0575 |
| Regional lymph node (N+) | 1.314 (1.031–1.675) | 0.0271 * | 1.308 (0.935–1.830) | 0.1167 |
| Stage (stage III & IV) | 1.016 (1.002–1.030) | 0.0289 * | 0.995 (0.969–1.021) | 0.6972 |

HNSCC, head and neck squamous cell carcinoma; HR, hazard ratio; CI, confidence interval; FAT1-HR, FAT1-associated high risk

**p*<0.05

**Supplementary Table 6.** Association of FAT1 signature with clinical and pathological features in five independent HNSCC cohorts (n=1079)

| Characteristics | FAT1-LR subgroup  (n=373) | FAT1-HR subgroup  (n=706) | p-value |
| --- | --- | --- | --- |
| Age |  |  |  |
| ≥60 | 47.85% | 54.33% | 0.0501 |
| <60 | 52.15% | 45.67% |  |
| Sex |  |  |  |
| Male | 80.43% | 73.09% | 0.0094 * |
| Female | 19.57% | 26.91% |  |
| Smoking |  |  |  |
| Yes | 78.64% | 77.14% | 0.6528 |
| No | 21.36% | 22.86% |  |
| Alcohol |  |  |  |
| Yes | 74.23% | 74.06% | 1 |
| No | 25.77% | 25.94% |  |
| Tumor site |  |  |  |
| Oral cavity | 44.47% | 67.47% | 2.2e-16 * |
| Oropharynx | 31.54% | 11.51% |  |
| Larynx | 18.87% | 17.61% |  |
| Hypopharynx | 5.12% | 3.41% |  |
| Stage |  |  |  |
| I-II | 25.21% | 26.69% | 0.6558 |
| III-IV | 74.79% | 73.31% |  |
| HPV status |  |  |  |
| Positive | 36.21% | 7.09% | 4.885e-15 * |
| Negative | 63.79% | 92.91% |  |
| Radiotherapy |  |  |  |
| Yes | 70.1% | 60.63% | 0.02683 * |
| No | 29.9% | 39.37% |  |

HNSCC, head and neck squamous cell carcinoma; FAT1-LR, FAT1-associated low risk; FAT1-HR, FAT1-associated high risk. **p*<0.05

**Supplementary Table 7.** Analysis of mRNA expression of the 23 FAT1-associated signature genes and FAT1 gene in the KHU HNSCC cohort ^a^

| Gene symbol | Gene name | Genetic significance with non-recurrence or recurrence of HNSCC patients after radiotherapy (*p*-value) | |  |
| --- | --- | --- | --- | --- |
| NRG1  TCEA3  PCDHGA10  FER1L6  PCDH7  FAM3B  SLC16A8  TFPI2  SYNE4  MT3  DSCAM  KEL  SEMA3A  F2RL1  SYNGR1  FUZ  NKX2-3  AS3MT  SLC7A11  SYCE2  BEX2  BRME1  CYP2E1  FAT1 | neuregulin 1  transcription elongation factor A3  protocadherin gamma subfamily A, 10  fer-1-like 6  protocadherin 7  family with sequence similarity 3 member B  solute carrier family 16 member 8  tissue factor pathway inhibitor 2  spectrin repeat-containing nuclear envelope family member 4  metallothionein 3  DS cell adhesion molecule  Kell blood group, metallo-endopeptidase  semaphorin 3A  F2R like trypsin receptor 1  synaptogyrin 1  fuzzy planar cell polarity protein  NK2 homeobox 3  arsenite methyltransferase  solute carrier family 7 member 11  synaptonemal complex central element protein 2  brain expressed X-linked 2  break repair meiotic recombinase recruitment factor 1  cytochrome P450 family 2 subfamily E member 1  FAT atypical cadherin 1 | 0.2202  0.0031  0.0493  0.2158  0.0499  0.2153  0.4106  0.2168  0.3492  0.1848  0.0128  0.0422  0.0246  0.0081  0.0061  0.0624  0.2370  0.0628  0.1344  0.3050  0.2506  0.2139  0.0081  0.0294 | NS  **  *  NS  *  NS  NS  NS  NS  NS  *  *  *  **  **  NS  NS  NS  NS  NS  NS  NS  **  * | |

HNSCC, head and neck squamous cell carcinoma

^a^ An independent samples *t*-test was used to compare the values between the two groups. * means *p*<0.05, ** means *p*<0.01.
