# Supplementary material for: Clinical significance of FAT1 gene mutation and mRNA expression in patients with head and neck squamous cell carcinoma
Source: Mol Oncol. 2022 Jan 13;16(8):1661–79. doi: 10.1002/1878-0261.13171 (PMC9019907; doi:10.1002/1878-0261.13171)
Supplement: Supplementary file 7 — Appendix S1. Real‐time quantitative reverse transcriptase PCR analysis. [file MOL2-16-1661-s004.docx]

**Supplementary materials and methods**

*Real-time quantitative reverse transcriptase PCR analysis*

Total RNA was extracted using the TRIzol^®^ reagent method (Invitrogen, Carlsbad, CA, USA) as previously described.^20^ In more detail, frozen tissues were homogenized in 1 mL TRIzol^®^ reagent and briefly vortexed, following which 200 μL chloroform was added to the samples. The mixtures were centrifuged for 15 min at 12,000 × *g* at 4°C, and the mixture was separated into a lower red phenol-chloroform interphase and a colorless upper aqueous phase. The supernatant containing the RNA was then transferred to a new tube. RNA precipitation was performed with 500 μL isopropanol, and the RNA pellets were washed with 1 mL of 70% ice-cold ethanol.

Total RNA was reverse transcribed to cDNA using a Tetro cDNA Synthesis Kit (Bioline, USA), according to the manufacturer’s recommended protocol. Real-time quantitative PCR was performed using the SensiFAST™ SYBR Hi-ROX Kit (Bioline) with specific primers. All real-time quantitative PCR experiments were performed in triplicate, and quantification cycle (Cq) values were determined using StepOne Software v2.3. Relative quantification of mRNA levels was performed using the comparative Ct method, with β-actin as the reference gene. The following primer sets were used for real-time PCR experiments: β-actin, 5′-GGACTTCGAGCAAGAGATGG-3′ (forward) and 5′-AGCACTGTGTTGGCGTACAG-3′ (reverse); FAT1, 5′- CCTTCCAACAGCCACATCCACTAC-3′ (forward) and 5′-TTGAACCGTGAGCGTGTAACCTG-3′ (reverse). All experiments were performed in triplicate and the values were averaged.
