# Supplementary material for: Clinical significance of FAT1 gene mutation and mRNA expression in patients with head and neck squamous cell carcinoma
Source: Mol Oncol. 2022 Jan 13;16(8):1661–79. doi: 10.1002/1878-0261.13171 (PMC9019907; doi:10.1002/1878-0261.13171)
Supplement: Supplementary file 8 [file MOL2-16-1661-s006.docx]

**Supplementary Figure Legends**

**Supplementary Fig. 1.** FAT1 alteration in HNSCC. (A) Cross-cancer summary of FAT1 alteration in 31 cancer types using mutation and copy number data obtained from the TCGA cohort. HNSCC was the first cancer to show FAT1 alteration (mutation=20.08%, fusion=0.19%, amplification=0.57%, deep deletion=5.54%, and multiple alteration=1.15%). (B) The plot showing the proportion of FAT1 mutation types in TCGA HNSCC cohort (truncating=79.28%, missense=16.22%, splice=3.60%, and inframe=0.90%).

**Supplementary Fig. 2.** FAT1 mRNA expression and mutation status in the TCGA HNSCC patients. (A) FAT1 mRNA expression was significantly lower in FAT1 mutated patients than FAT1 wild-type patients (*p*=4.975e-05). (B) An ROC curve analysis of FAT1 mRNA expression levels was performed to distinguish between FAT1 wild-type and mutated patients. Youden-index was maximized at the cut-off value of FAT1 mRNA expression >12.9071 (hereafter referred to as the Youden-index threshold). (C, D) Five-year OS and RFS rates of FAT1 low and high expression subgroups, based on the Youden-index threshold in TCGA HNSCC patients, were depicted using Kaplan-Meier plots. (E, F) In the same way, five-year OS and RFS rates of FAT1 wild-type and mutated subgroups were depicted in TCGA HNSCC patients. There were no significant differences in five-year OS and RFS rates between FAT1 low and high expression subgroups. Also, there were no significant differences in five-year OS and RFS rates between FAT1 wild-type and mutated subgroups. **p*<0.05

**Supplementary Fig. 3.** Construction of the prediction model. (A) Venn diagram showing number of gene signatures correlated with FAT1 mRNA expression and mutation. (B) Schematic overview of the strategy used for constructing prediction models and evaluating the predicted outcomes based on gene expression signatures.

**Supplementary Fig. 4.** FAT1 is a crucial factor for maintenance repair of radiotherapy-induced DNA damage in FAT1-HR HNSCC cell lines. (A) A total of 15 HNSCC cell lines were classified into FAT1-LR and FAT1-HR subgroups using an algorithm based on BCCP. (B) FAT1 and β-actin mRNA levels were determined using qRT-PCR analysis. Graphs represent three independent experiments performed in triplicate. Error bars represent standard deviations from the mean. ***p*<0.001 and ****p*<0.0001. DNA damage to FAT1-HR HNSCC cell lines such as HSC3 and YD38 upon radiation exposure was assessed using ICC. HSC3 and YD38 were transfected with siGFP, siFAT1 #1 or siFAT1 #2 before radiation exposure at 0 or 4 Gy. (C) The cells were stained for γ-H2AX (green) and DNA (blue; DAPI), and observed using confocal microscopy. (D) The foci distributions were titrated on the same sample images. Scale bar is 50μm

**Supplementary Fig. 5.** FAT1 modulated radiation sensitivity in the radioresistant NHSCC cell line. (A) The FAT1 protein levels in the siGFP-transfected CAL27-P, siGFP-transfected CAL27-RR, and siFAT1 #2-transfected CAL27-RR cells were determined using western blot, to verify the status of FAT1 protein level. β-Actin was included as an internal loading control. The radiation susceptibility was estimated using colony-forming assay. CAL27-P and CAL27-RR were transfected with siGFP and siFAT1 #2, respectively. (B) The cells underwent colony formation under radiation doses of 0, 2, 4, and 8 Gy. All the cells were repeated 3 times; the figure shows a representative image upon staining with Crystal Violet after about 10 d.
